# Comprehensive lincRNA Transcriptome in Acute Myeloid Leukemia: Integrating Known and Newly Identified lincRNAs Across Pediatric and Adult Cohorts

**DOI:** 10.3390/ncrna12030018

**Published:** 2026-05-27

**Authors:** Saioa Arza-Apalategi, Daan Gilissen, Anne C. van der Grinten, Seline N. van den Oever, Erik B. van den Akker, Marieke Griffioen, Joop H. Jansen, Joost H. A. Martens, Anna E. Marneth, Bert A. van der Reijden

**Affiliations:** 1 Department of Laboratory Medicine, Laboratory of Hematology, Radboud Institute for Medical Innovation, Radboud University Medical Center, 6500 HB Nijmegen, The Netherlands; 2 Department of Biomedical Data Sciences, Leiden University Medical Center, 2333 ZA Leiden, The Netherlands; 3 Pattern Recognition & Bioinformatics, Delft University of Technology, 2628 XE Delft, The Netherlands; 4 Leiden Center for Computational Oncology, Leiden University Medical Center, 2333 ZA Leiden, The Netherlands; 5 Department of Hematology, Leiden University Medical Center, 2333 ZA Leiden, The Netherlands; 6 Department of Molecular Biology, Faculty of Science, Radboud University, 6525 GA Nijmegen, The Netherlands

**Keywords:** acute myeloid leukemia, transcript discovery, multi-cohort, bioinformatics, RNA-Seq

## Abstract

**Background/Objectives**: Acute myeloid leukemia (AML) comprises genetic subclasses with distinct gene expression profiles. While AML gene expression studies have mainly focused on protein-coding genes, our understanding of expression patterns of long intergenic noncoding RNAs (lincRNAs) remains incomplete. This is due to limited sample sizes, as well as incomplete annotation of lncRNAs with context-dependent expression. **Methods**: To address this gap, we developed the bioinformatic pipeline LIRA (long intergenic noncoding RNA annotator) to identify novel lincRNAs using stringent criteria, including spliced and intergenic transcripts, and algorithms to exclude coding potential. **Results**: By applying LIRA to RNA-sequencing data from 878 pediatric and adult AML cases and 20 healthy controls, we identified 1560 novel lincRNAs, expanding the GENCODE v38 lincRNA catalog by 27%. Integration of in-house-generated CAGE- and ChIP-sequencing data from KMT2A::MLLT3 samples revealed that 80% of the novel lincRNAs are 5′ capped, and at least 67% harbor activating epigenetic marks at their transcription start sites. Unsupervised analysis of the 1000 most variable known and newly identified lincRNAs uncovered subclass-specific expression patterns, mirroring those observed for protein-coding genes. Weighted Gene Co-expression Network Analysis identified 17 lincRNA expression modules associated with AML subclasses. Notably, expression of these modules decreased upon degradation of the leukemogenic onco-fusion proteins KMT2A::MLLT3 and PML::RARA, indicating that lincRNA expression is responsive to oncogenic signaling. **Conclusions**: This comprehensive analysis shows that lincRNAs exhibit similar subclass-specific expression patterns as protein-coding genes and establishes a valuable resource for future studies on genetically defined AML subclasses, with potential implications for biomarker discovery and therapeutic targeting.

## 1. Introduction

Acute myeloid leukemia (AML) is a genetically heterogeneous disease characterized by the expansion of immature blood cells in the bone marrow that suppress the formation of healthy blood cells. For disease classification and risk stratification, several AML classes are recognized, which are defined by the presence of specific genetic aberrations. These drivers are often transcriptional or epigenetic regulators, which disrupt programs controlling differentiation, proliferation and apoptosis. Several studies have shown that AML can also be classified based on gene expression profiles that associate with recurrent genetic mutations [1,2,3,4,5]. While most genetically defined AML subclasses have a unique gene expression profile, various subclasses can be divided into multiple transcriptionally defined clusters [1,3,6]. For example, in NPM1- and KMT2A::MLLT3-mutated AML, five and two gene expression profiles were described, respectively, which could not be explained by recurrent co-mutations. Further insight into transcriptional changes in AML is pursued by studying the marker genes that characterize these unique gene expression profiles. Remarkably, long noncoding RNAs (lncRNAs) are often found as marker genes, such as UCA1 for CEBPA-mutated cases, MEG3 in PML::RARA-mutated cases and LINC01978 in one of the NPM1-mutated profiles [3,4,7,8]. LncRNAs are defined as RNAs longer than 200 nucleotides that are not translated into proteins. LncRNAs play crucial roles in many biological processes, such as gene expression regulation, metabolism, and signaling [9]. Changes in their expression have been implicated in cancer development, including in AML [10,11,12,13,14]. Indeed, based on the expression of a small set of lncRNAs, prediction models for survival [15], prognosis [7,16,17,18] or relapse [18,19] have been developed for specific groups of AML. Yet, a comprehensive identification of lncRNA expressed in AML and their association with distinct genetic abnormalities in large patient cohorts are lacking. This is partly due to their tissue-specific expression patterns and the challenge in identifying and functionally characterizing these RNA molecules. Additionally, the identification and functional characterization of lncRNAs have largely been focused on a healthy context, not taking into consideration that leukemia-specific lncRNA expression occurs [20]. To address this knowledge gap, we set out to identify novel intergenic lncRNAs (lincRNAs) from large patient cohorts at an unprecedented scale [21]. Therefore, we developed a bioinformatic pipeline called long intergenic noncoding RNA annotator (LIRA) to identify unannotated lincRNAs using RNA-sequencing data of 878 AML samples and 20 healthy reference samples (from the pediatric AML-05 and TARGET and the adult BEAT and TCGA AML cohorts) [22,23,24,25]. This identified 1560 previously unannotated lincRNAs. These, together with the 5858 known lincRNAs (GENCODE v38) were used to define the association between the lincRNA transcriptome and genetically defined AML subclasses.

## 2. Results

### 2.1. Long Intergenic Noncoding RNA Annotator (LIRA) Identifies 1560 Previously Unannotated lincRNAs

**Figure 1:**
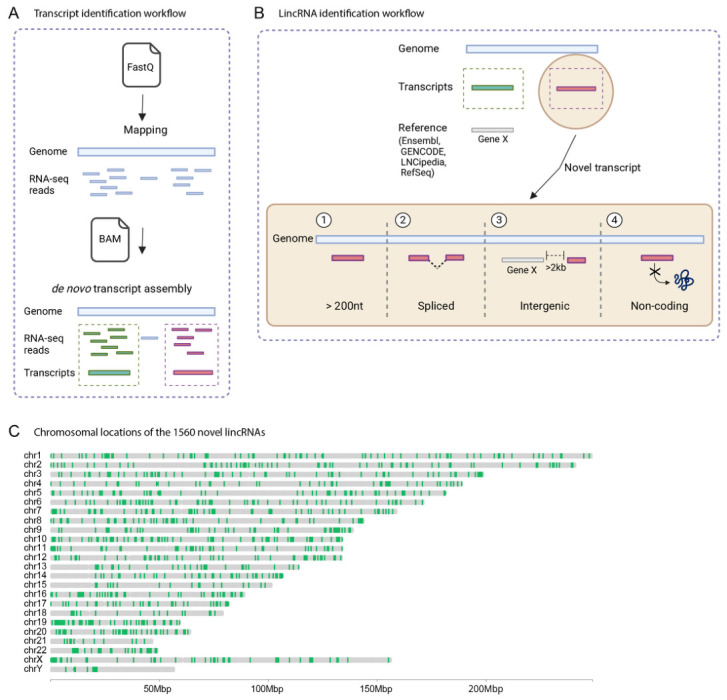
Using 878 AML and 20 healthy samples, LIRA identified 1560 previously unannotated lincRNAs. (**A**) Schematic representation of the transcript identification workflow used by LIRA. Bulk RNA-seq data (FastQ files) were mapped to the reference genome using STAR, and BAM files were generated. De novo transcript assembly was performed on BAM files of the 898 samples to construct transcripts using StringTie. (**B**) Filtering steps for identifying novel lincRNAs. After transcript identification, all transcripts overlapping with previously annotated (Ensembl, GENCODE, LNCipedia and RefSeq) genes were excluded. The remaining transcripts were filtered based on length (>200 nt), splicing, genomic location (intergenic), and lack of coding potential (based on CPAT and PLEK) to identify novel lincRNAs. (**C**) Chromosomal distribution of the 1560 newly identified lincRNAs across the four cohorts. This graph was made using R (version 4.5.3).

We hypothesized that AML mutations may result in expression of lincRNAs that have not been identified in healthy tissues. To study the presence of previously unannotated lincRNAs in RNA-seq data, we developed a bioinformatic pipeline called LIRA, as visualized in Figure 1A,B. High-quality RNA reads of 898 samples (878 AML, 19 healthy individual bone marrow and 1 healthy individual CD34+) from four independent cohorts were mapped to the GRCh38 reference genome using STAR [26], and transcriptome assembly was performed with StringTie [27]. To identify novel lincRNAs in our AML and healthy samples, we first excluded all previously annotated transcripts from Ensembl, GENCODE, LNCipedia and RefSeq. To mitigate the impact of technical constraints and minimize the likelihood of false-positive identifications, our investigation was centered on long intergenic noncoding RNAs that did not overlap with annotated transcripts. In addition, as the detection of multiple junction reads increases the likelihood of finding true transcripts, we excluded non-spliced transcripts. Given the incomplete characterization of the untranslated regions (UTRs) in numerous genes, transcripts overlapping with DNA regions within 2000 base pairs downstream or upstream of annotated genes were systematically excluded from our analysis. Lastly, the coding potential of the newly identified transcripts was assessed using CPAT and PLEK [28,29]. Transcripts with coding potential in either tool were filtered out. Using these stringent criteria, we confidently identified 1560 novel lincRNAs (LIRA1-1560) (Table S1). Figure 1C shows that the novel lincRNAs are spread across the entire genome, with expected gaps on the gene-poor short arms of chromosomes 13, 14, and 15 and the long arm of the Y chromosome [30]. The lincRNAs had fewer exons compared to protein-coding transcripts, and they are smaller in size (Figure S1) [31]. In the final step of LIRA, read counts were obtained for protein-coding genes (Ensembl, *n* = 19,963), lincRNA (GENCODE, *n* = 5858) and the 1560 novel lincRNAs, which were used for further analysis (Tables S2 and S3).

### 2.2. Novel lincRNAs Are 5′ Capped and Their Transcription Start Sites Contain Transcription-Associated Epigenetic Marks

**Figure 2:**
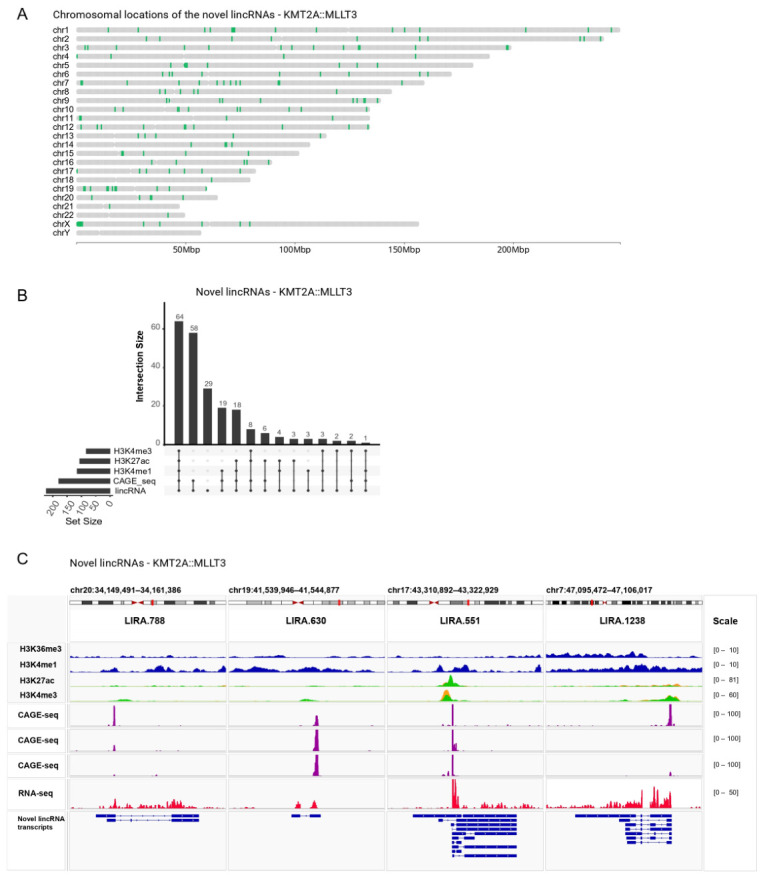
Validation of novel lincRNAs expressed in KMT2A::MLLT3 AML samples. (**A**) Chromosomal distribution of 220 novel lincRNAs expressed based on 6 KMT2A::MLLT3 samples. Graph made with R. (**B**) Upset plot showing the overlap between the genomic annotations of the 220 lincRNAs and epigenetic marks (H3K4me1, H3K27ac, H3K4me3) derived from ChIP-seq data and transcription start site peaks based on unidirectional CAGE-seq. The CAGE-seq data are derived from 6 KMT2A::MLLT3 AML samples [6], while histone mark data originate from the BLUEPRINT consortium [34]. (**C**) IGV visualization of the genomic loci of selected novel lincRNAs (LIRA.788, LIRA.630, LIRA.551, LIRA.1238) with associated RNA-seq coverage, epigenetic marks (H3K36me3, H3K4me1, H3K27ac, H3K4me3), and transcription start sites based on CAGE-seq data. This panel provides a detailed representation of the regulatory landscape at these loci. The H3K27ac and H3K4me3 tracks represent data from two individuals, shown in green and orange.

Most known lincRNAs are transcribed by RNA-polymerase II, which is directed by histone marks, such as H3K4me3, H3K4me1 and H3K27ac, that are frequently observed in active promoters. To study whether the novel lincRNAs are also associated with these marks, we interrogated independent in-house-generated ChIP-, RNA- and CAGE-seq (Cap Analysis of Gene Expression-seq) datasets of six KMT2A::MLLT3 AML samples [6,32,33]. RNA-seq analysis showed that 220 of the 1560 novel lincRNAs were expressed in these samples, and they were evenly distributed across the genome (Figure 2A). Unidirectional transcription start sites (TSSs) were determined by CAGE-seq and histone marks by ChIP-Seq. Of the 220 lincRNAs, 80% had overlap with a unidirectional TSS, from which 67% also showed overlap with at least one of the three histone marks indicative for active genomic regions (Figure 2B). In addition, we observed alternative TSSs, alternatively spliced lincRNAs and expression from both DNA strands. Some examples showcasing this heterogeneity are depicted in Figure 2C. The here reported similarities between novel and previously annotated lincRNAs strongly suggest that the newly identified ones represent true lincRNAs.

### 2.3. LincRNAs Exhibit Distinct Expression Patterns in Genetically Defined AML Subclasses

**Figure 3:**
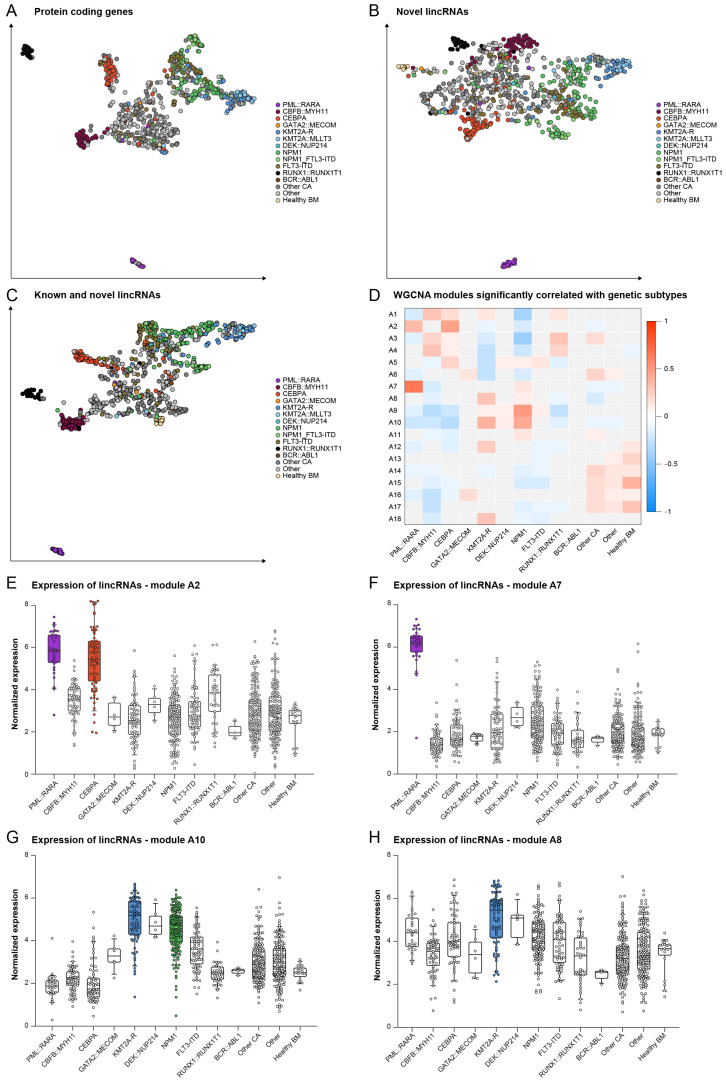
LincRNA-based grouping of AML samples and module correlation with genetically defined subtypes. (**A**–**C**) UMAP visualizations showing the clustering of 878 AML samples and 19 healthy bone marrow (BM) samples based on different datasets: (**A**) protein-coding gene expression data, (**B**) novel lincRNA expression data, and (**C**) combined known and novel lincRNA expression data. Each point represents a sample, and colors denote specific genetically defined AML subtypes or healthy BM. UMAPs for the separate cohorts can be found in Figure S2. The number of samples included in each subgroup are as follows: PML::RARA = 32, CBFB::MYH11 = 63, CEBPA = 73, GATA2::MECOM = 6, KMT2A-R = 45, KMT2A::MLLT3 = 29, DEK::NUP214 = 6, NPM1 = 108, NPM1_FLT3-ITD = 50, FLT3-ITD = 70, RUNX1::RUNX1T1 = 41, BCR::ABL1 = 3, Other CA = 177, Other = 175, Healthy BM = 19. (**D**) Heatmap illustrating the correlation between the overall expression pattern of genes in each WGCNA module and the presence of specific genetic subtypes (*X*-axis). Modules with stronger correlations are represented by more intense colors, ranging from negative (blue) to positive (red). Gray color represents non-significant (*p* > 0.01) correlations. KMT2A-R group contains all KMT2A-rearrangements, including KMT2A::MLLT3. NPM1 group contains all samples classified as NPM1 mutant, irrespective of FLT3-ITD presence, while the FLT3-ITD group contains samples that are positive for FLT3-ITD and wild type NPM1. Module A18 contains the lincRNAs that are not co-expressed in a module. (**E**–**H**) Boxplots showing the mean variance of stabilized normalized expression of lincRNAs in specific WGCNA modules (modules 2, 7, 10, and 8) across various genetic subtypes. Subtypes that have a strong positive correlation with the respective modules in (**D**) are shown in color. Each boxplot illustrates the expression variability within a genetic subtype, highlighting module-specific lincRNA associations with genetic aberrations.

Our next goal was to determine whether genetically defined AML subclasses exhibit distinct lincRNA expression patterns as previously described for protein-coding genes [3,4,5]. Following data integration and batch correction of the four cohorts, we performed Uniform Manifold Approximation and Projection (UMAP) analysis on the 1000 most variable protein-coding transcripts and compared this to the 1000 most variable novel lincRNAs. As anticipated [3,12], strong protein-coding transcriptional similarities were observed for distinct genetic subclasses, such as the CBFB::MYH11-, PML::RARA-, KMT2A::MLLT3-, RUNX1::RUNX1T1- and NPM1-mutated subclasses (Figure 3A, with individual cohorts presented in Figure S2). The UMAP of the 1000 most variable novel lincRNAs showed very similar results as those observed for protein-coding RNAs (Figure 3B and Figure S2). To determine the association between genetically defined AML subclasses and all lincRNAs, we integrated the novel (21%) and known lincRNAs (79%) and repeated the UMAP analysis using the 1000 most variable lincRNAs. Of these 1000 lincRNA, 35% were novel. Again, samples displayed clear grouping according to their genetically defined AML subclass (Figure 3C). Together, these data indicate that the genetic makeup of AML strongly associates with both protein-coding and lincRNA expression profiles.

### 2.4. LincRNA Co-Expression Modules Associated with Genetically Defined AML Subclasses

To identify modules of highly interconnected genes that associate with genetically defined AML subclasses, we first performed Weighted Gene Co-expression Network Analysis (WGCNA) on the same set of 1000 most variable lincRNAs used to generate Figure 3C. This identified 17 modules with co-expressed lincRNAs and 1 module with the remainder of the lincRNAs (Figure 3D). The modules with co-expressed lincRNAs varied in size (10–183 genes) and percentage of novel lincRNAs (13–92%) (Figure S3, Tables S4 and S5). Subsequently, we assessed the correlation between the co-expressed genes in these modules and genetically defined AML subclasses or healthy BM. This showed that every module exhibited a significant positive (red) or negative (blue) correlation with at least three specific subclasses (Figure 3D). Genes in modules with a strong positive subclass correlation were also highly expressed in that subclass, as exemplified for modules correlated with KMT2A rearrangements (KMT2A-R) and PML::RARA (Figure 3E–H). Although most lincRNAs have not yet been characterized, the identified modules contain 32 known lincRNA implicated in normal and malignant hematopoiesis (Table S6). Thus, we were able to both recapture findings from previous smaller lincRNA studies and identify novel lincRNAs of interest.

### 2.5. Subclass-Specific lincRNA Expression Diminishes upon KMT2A::MLLT3 and PML::RARA Degradation

**Figure 4:**
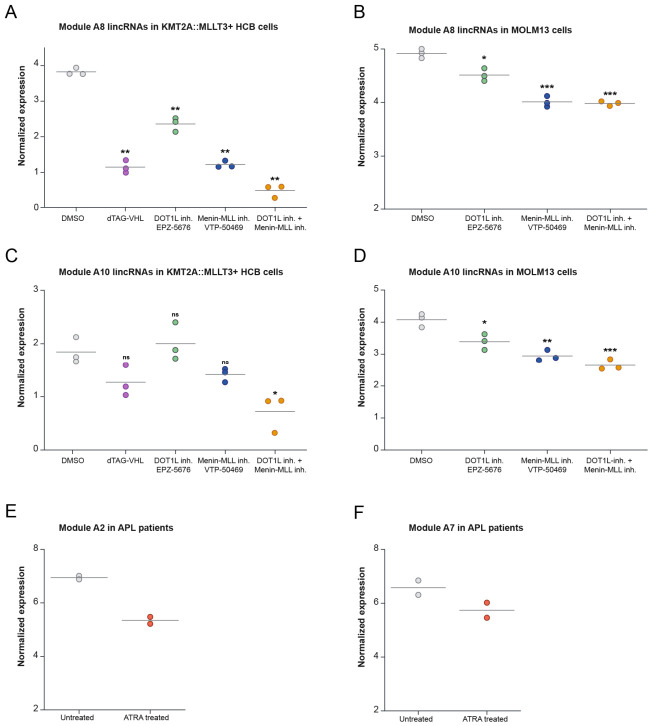
Downregulation of lincRNAs in response to KMT2A::MLLT3 degradation or ATRA treatment. (**A**–**D**) Normalized expression of lincRNAs from modules 8 and 10 in KMT2A::MLLT3-positive HCB cells ((**A**) and (**C**)**,** respectively) and MOLM13 cells ((**B**) and (**D**), respectively) under various treatments. Treatments include DMSO (control), dTAG-VHL (degronTAG-von Hippel-Lindau) for KMT2A::MLLT3 degradation (in KMT2A::MLLT3+ HCB cells), DOT1L inhibition (EPZ-5676), MENIN inhibition (VTP-50469), and combined DOT1L and MENIN inhibition. (**E**,**F**) Expression of lincRNAs from modules 2 and 7 in acute promyelocytic leukemia patients with PML::RARA translocation. Expression levels are compared between untreated and ATRA-treated conditions. Statistical significance in A–D is calculated using one-way ANOVA with Holm–Šídák multiple comparisons correction, denoted by *p* values: ns (not significant), *p* < 0.05 (*), *p* < 0.01 (**), and *p* < 0.001 (***). The asterisks above each condition represent the statistical significance between the condition and the control.

Previous studies have shown that protein-coding gene expression programs depend on onco-fusion proteins such as KMT2A::MLLT3 and PML::RARA [35,36]. Here we studied whether high lincRNA module expression also depends on these onco-fusion proteins. PROTAC-mediated onco-fusion protein degradation in primary cord blood CD34+ KMT2A::MLLT3-immortalized cells resulted in significantly lower expression of modules 8 and 10 (Figure 4A,C). Similar results were obtained when these cells or KMT2A::MLLT3+ MOLM-13 cells were treated with small-molecule DOT1L and MENIN (MEN1) inhibitors that reduce KMT2A::MLLT3-induced gene expression. Upon co-treatment of these inhibitors, the downregulation of lincRNA module expression was stronger compared to single-agent treatment, as was observed for protein-coding genes [35] (Figure 4A–D). This indicates that expression of these lincRNA modules depends on KMT2A::MLLT3 activity. Secondly, we interrogated RNA-seq data of bone marrow samples from two PML::RARA-positive patients before and after all-trans retinoic acid (ATRA) treatment in vitro [36], which triggers degradation of the onco-fusion protein [37]. This showed a downward trend in expression of modules 2 and 7 following ATRA treatment (Figure 4E,F). This analysis indicated once more a link between the fusion protein and the expression of a specific set of lincRNAs.

### 2.6. Differences in lincRNA Co-Expression Modules Between Pediatric and Adult AML

**Figure 5:**
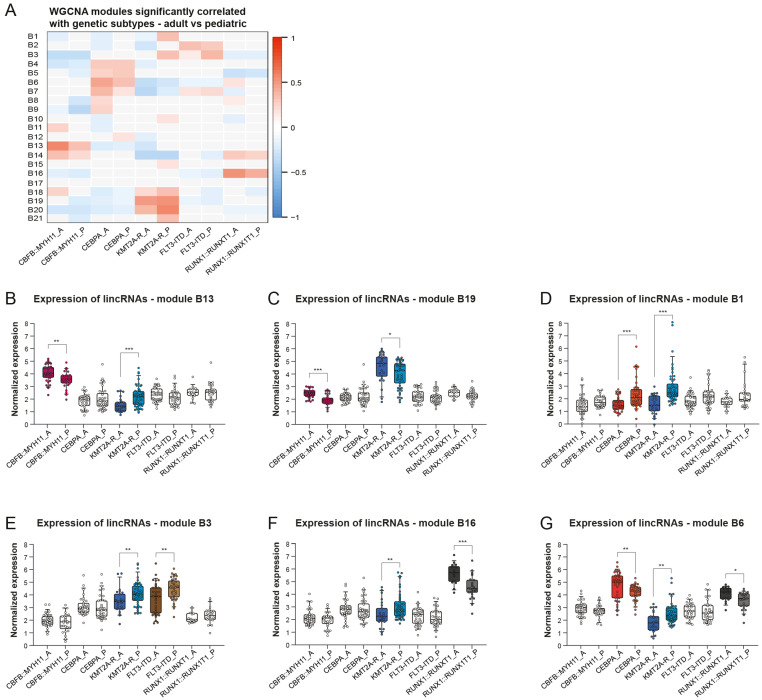
Module expression differences between adult and pediatric AML samples across genetic subtypes. (**A**) Heatmap showing the correlation between WGCNA modules and genetic subtypes in adult and pediatric AML samples. The scale represents correlation coefficients, with blue indicating negative correlations and red indicating positive correlations. Gray color represents non-significant (*p* > 0.01) correlations. The number of samples included in each subgroup are as follows: CBFB::MYH11_A = 35, CBFB::MYH11_P = 28, CEBPA_A = 34, CEBPA_P = 39, KMT2A-R_A = 25, KMT2A-R_P = 49, FLT3-ITD_A = 35, FLT3-ITD_P = 35, RUNX1::RUNX1T1_A = 16, RUNX1::RUNX1T1_P = 25. (**B**–**G**) Boxplots illustrating the normalized expression levels of lincRNAs in selected WGCNA modules across various genetic subtypes in adult and pediatric AML samples. Significant differences in expression between adult (_A) and pediatric (_P) samples are observed for specific subtypes. Boxplots with significant differences between age categories are highlighted in color. Statistical significance is denoted as *p* < 0.05 (*), *p* < 0.01 (**), and *p* < 0.001 (***).

To elucidate whether disparities exist among AML patients of different age categories, we conducted a new WCGNA focusing on a subset of samples with frequently occurring genetic abnormalities shared between pediatric and adult AML, including CEBPA or FLT3 ITD mutations and CBFB::MYH11, RUNX1::RUNX1T1 and KMT2A rearrangements (Table S7). WGCNA identified 20 modules with co-expressed lincRNAs and 1 module with the remainder of the lincRNAs (Figure 5A and Figure S2, Tables S8 and S9). Most modules exhibited a significant correlation with at least two specific subclasses irrespective of age. Approximately 50% of these correlations were present in both pediatric and adult AML (Figure 5A). The boxplots in Figure 5B–G confirm the correlations observed in the heatmap (Figure 5A), but they also indicate the expression differences within the same subclass between adult and pediatric cases. Although expression levels of lincRNAs show clear differences between pediatric and adult AML, in general, the expression patterns are mainly associated with genetic subclasses irrespective of age.

## 3. Discussion

Previous research has mostly focused on the identification of lincRNAs in healthy tissues [20]. In this study, we developed a pipeline named LIRA to annotate lincRNAs and identified 1560 previously unannotated lincRNAs in 898 samples (878 AML and 20 healthy samples), expanding the number of known lincRNAs by 27%. These novel lincRNAs likely reflect both AML-associated transcriptional dysregulation and more ubiquitously expressed transcripts that remained unannotated due to low expression or limited sampling of specific cell types and developmental stages. GENCODE v47, which is the latest version and which was released during the generation of this manuscript, shows overlap for 590 of our novel lincRNAs, further validating LIRA’s ability to discover lincRNAs. We expect that more lncRNAs will be identified in the future, as our pipeline deliberately focused on the detection of spliced intergenic lncRNAs to avoid the risk of identifying false positives. Consequently, gene overlapping (sense or antisense) and intronic lncRNAs such as ANRIL, RUNXOR, IRAIN and HOTAIRM1 were not considered here [11]. Functionally, this focus likely led to an enrichment of independently transcribed loci while excluding most lncRNAs with cis-regulatory or antisense functions. Novel technologies like long-read RNA-seq may more reliably identify gene-overlapping lncRNAs, as they are able to identify full-length transcripts without the need for assembly and thus have fewer artifacts compared to short-read RNA-seq [38]. Nevertheless, our freely available LIRA pipeline can be used to discover the total lincRNA landscape in any biological context in this largely uncharted field.

The large number of samples and identification of additional spliced lincRNAs allowed us to present what is to our knowledge the most comprehensive overview of lincRNA expression in AML so far. AML cases with recurrent genetic abnormalities grouped robustly together tightly in unsupervised UMAP analyses based on lincRNA expression, similar to protein-coding genes. This is an important finding considering the regulatory functions of lincRNAs, highlighting the relevance of studying lincRNA expression patterns in AML. Furthermore, we identified sets of co-expressed lincRNAs (modules) that associate with various genetically defined AML subclasses and age categories. Some modules showed high expression in multiple genetically defined subclasses. Rather than focusing on differences in the expression of individual lincRNAs, this approach captures coordinated gene expression patterns. While many modules consistently associated with genetically defined subclasses regardless of age, we did observe significant differences in mean module expression between age categories. This indicates that subtype-driven lincRNA expression patterns are broadly conserved. However, separate analysis of pediatric and adult cases may be advised in applications where expression levels are critical, such as biomarker discovery.

When including all samples, we identified 17 modules that associated with various genetically defined AML subclasses. These findings serve as a resource to guide future studies, including targeted experimental validation of prioritized lincRNAs and functional characterization in independent cohorts. Here, we used two independent patient datasets and showed that high mean expression of various lincRNA modules was normalized upon degradation of leukemia-driving onco-fusion proteins KMT2A::MLLT3 and PML::RARA. This suggests that high module expression is a downstream effect of these onco-fusion proteins. It will be relevant to determine whether individual lincRNA expression is directly activated by the onco-fusion proteins.

The 17 modules included a total of 755 different lincRNA. In the literature, we found associations with leukemia for 32 of these lincRNAs (Table S6) [3,8,14,15,16,21,39,40,41,42,43,44,45,46,47,48,49,50,51,52,53,54,55,56,57,58,59,60,61,62,63,64]. For example, PML::RARA-associated module A7 included CRNDE. This lincRNA was found to be overexpressed in PML::RARA and is thought to be involved in the disease pathobiology [65]. Overexpression of PCAT18 (module A8) was previously described in AML patients with an NPM1 mutation and was found to accelerate G1/S cell cycle transition and proliferation [48]. Finally, nanoparticle-mediated silencing of LINC01257, present in module A4, showed promise in preclinical RUNX1::RUNX1T1 pediatric AML studies [41]. Altogether, the functions of many lincRNAs have not yet been described. However, their association with AML incentivizes further study of their roles in AML, as well as their potential as biomarkers and therapeutic targets, with the ultimate goal of improving patient outcomes.

## 4. Materials and Methods

### 4.1. Data Acquisition

BAM files of BEATAML1.0 (*n* = 430), TARGET-AML (*n* = 178) and TCGA-LAML (*n* = 151) were acquired from GDC (https://portal.gdc.cancer.gov/) on 9 February 2022. Access to AML-05 transcriptomics data (*n* = 139) was provided by the AML-05 study group at Kyoto University, Department of Pathology and Tumor Biology (see acknowledgments). These samples are all primary AML samples (follow-up and relapse samples were excluded), except healthy individual samples from Beat AML (19 bone marrow samples and 1 CD34+ sample). Corresponding metadata about age, gender, gene mutations and cytogenetic aberrations was also obtained.

### 4.2. Data Preprocessing

BAM files from Beat AML and AML-05 were converted to paired-end FASTQ files using samtools (v1.15.1). The GRCh38 reference genome was indexed using STAR aligner (v2.7.9a) with Ensembl gene annotation version 95. For Beat AML and AML-05, paired-end reads were trimmed and checked for quality using Trim Galore (v0.6.6). The resulting trimmed reads were mapped against the indexed reference genome in two-pass mode with the same parameters as GDC mRNA Analysis Pipeline version Dr15plus.

### 4.3. LincRNA Detection Workflow LIRA

BAM files for each of the four cohorts were used as input for StringTie (v2.1.6) [27]. StringTie assembly was performed for each sample using a combined annotation of known genes from Ensembl (v95), RefSeq (release 213), GENCODE (v38) lincRNA and LNCipedia (v5.2) as reference, along with the following non-default parameters: --rf, -c 5, -f 0.1, -j 3. StringTie merge was used to create a consensus transcript annotation for each of the four cohorts individually. GFFcompare (v0.12.6) was used to determine novel intergenic transcripts by comparing the assembled annotation from StringTie with the combined annotation of known genes, filtering for intergenic transcripts (“u” transfrag). Sense and antisense lncRNAs were excluded due to the high risk of detecting false positives, as observed during the pipeline development process. Next, transcripts were filtered for a length of over 200 nucleotides. PLEK (v1.2) and CPAT (v3.0.4) were used to determine the coding potential of the identified transcripts. Both tools are alignment-free and classify transcripts as protein-coding or noncoding based on k-mer usage (PLEK) or a combination of sequence features (CPAT). Transcripts with no or low coding potential in both PLEK and CPAT (using default cutoffs) were kept for the novel lincRNA annotation per cohort. To minimize false positives, only transcripts with more than one exon were retained. Finally, novel transcripts within 2000 nucleotides from known genes were excluded to prevent overlap with untranslated regions (UTRs). StringTie merge was used to make a combined novel lincRNA annotation of the four cohorts. No restriction was placed on the minimum number of samples in which a transcript is expressed. For each sample, gene counts were produced for protein-coding genes (Ensembl, *n* = 19,963), known lincRNA (GENCODE v38 lincRNA, *n* = 5858) and novel lincRNA (*n* = 1560) using FeatureCounts (Subread v2.0.3).

### 4.4. Fusion Gene Detection

Fusion genes were determined for the Beat AML samples using FusionCatcher (v1.10) with paired-end fastq files as input and STAR-Fusion (v1.7.0) and Arriba (v1.1.0) using junction files from the STAR aligner as input. Overlap between the three fusion detection tools was determined using FuMa (v3.0.5). For each sample, the most reliable fusion gene was selected based on the number of tools that identified the fusion gene, whether a known fusion gene was found and the mean coverage of the fusion gene among the three tools. For the other three cohorts, the provided fusion gene annotations were used.

### 4.5. Processing 5′ Cap Analysis of Gene Expression (CAGE) Data

DNAFORM performed CAGE sequencing on an Illumina system and basic data preprocessing. Ribosomal RNA reads were removed from the fastq files using SortMeRNA (v4.3.4). The remaining reads were aligned against Ensembl GRCh38 primary assembly reference genome using BWA mem (v0.7.17-r1188). Reads with a mapping quality lower than 20 were aligned again using Hisat2 (v2.2.1). High-quality reads aligned by BWA mem and uniquely aligned reads by Hisat2 were merged into a single combined BAM file using samtools (v1.15.1). The Deeptools bamCoverage (v3.5.1) tool was used to make bigwig files for reads mapping on the forward and reverse strand separately. CAGEfightR (version 1.14.0) was used to analyze the bigwig files. The CAGE transcription start sites (CTSSs) were normalized (transcripts per million (TPM)), pooled, and pre-filtered with default settings. CTSSs within a range of 20 bp on the same strand were systematically clustered into unidirectional clusters.

### 4.6. Canonical Transcription Marks Overlapping with Novel lincRNAs in KMT2A::MLLT3+ AML Samples

RNA-seq data of 6 KMT2A::MLLT3+ AML samples were compared to CAGE data of 6 KMT2A::MLLT3+ AML samples and histone marks of 1 (H3K4me1) or 2 (H3K4me3, H3K27ac) samples from BLUEPRINT [6,33,34]. RNA-seq data were normalized and filtered with DESeq2 (v1.42.1), using a cutoff of RowSum > 100, RowMax > 45 and RowMean > 35. Overlap between lincRNA transcripts and CAGE unidirectional sites or histone peaks was determined using the chromosomal locations of the lincRNA transcripts plus and minus 500nt. UpsetR was used to plot and analyze the overlap between the different peaks and lincRNA transcripts. IGV (v2.16.0) was used to visually showcase the overlap between RNA-seq of novel lincRNA transcripts and CAGE and histone peaks.

### 4.7. LincRNA Transcriptomics Analysis

Protein-coding genes, novel lincRNAs and lincRNAs (novel combined with 5858 GENCODE lincRNAs) were separately analyzed as follows. Combat-seq (sva v3.50.0) was used for batch correction within cohorts (induction failure compared to others in TARGET and 3 sequencing batches in AML-05) and upon cohort integration. Corrected gene counts for each sample of the four cohorts were used as input for DESeq2 (v1.42.1). Variance stabilized transformation (VST) was used for transformation of normalized counts. UMAPs (umap v0.2.10.0) were made using the top 1000 most variable lincRNAs, based on the estimated variance obtained with the RowVars function. The same 1000 most variable genes were used for WGCNA (v1.72-5). The following non-default parameters were used in construction of the WGCNA network: networkType = “signed”, minModuleSize = 10, reassignThreshold = 0, mergeCutHeight = 0.25, deepSplit = 3, maxBlockSize = 25,000. Binary correlation matrices were made to determine correlation between expression of WGCNA modules of lincRNAs, based on the eigengene values, and genetically defined AML subclasses. Statistical analysis was conducted by calculating Student asymptotic *p*-value using corPvalueStudent (WGCNA). For the comparison between adult and pediatric AML, WGCNA was repeated with the same settings but only including samples with CEBPA mutations, FLT3 ITD, CBFB::MYH11, RUNX1::RUNX1T1, or KMT2A-R. For the binary correlation matrices, each genetically defined AML subclass was split into an adult and a pediatric group. For every module, the mean expression was compared between adult and pediatric samples of each genetic subclass using Wilcoxon signed-rank tests with Benjamini Hochberg multiple testing correction.

### 4.8. LincRNA Expression in Onco-Fusion Degradation Experiments KMT2A::MLLT3 and PML::RARA

Two publicly available RNA-seq datasets were obtained. The first included RNA-seq data from stem and progenitor cells isolated from human cord blood cells transduced with KMT2A::MLLT3 C-terminally FKBP12-tagged (*n* = 3), before and 5 days after the onco-fusion protein degradation via the dTAG molecule [35]. The same dataset contained RNA-seq data of KMT2A::MLLT3 inhibition in the transduced cord blood cells and in MOLM-13 cells by two small molecules upon incubation of 5 days. EPZ-5676 is used to catalytically inhibit DOT1L, whereas VTP-50469 is used to impair the protein–protein interaction between MENIN and KMT2A. The second dataset, containing RNA-seq data of bone marrow from 2 donors with a PML::RARA fusion, was obtained from BLUEPRINT [36]. The data included 2 replicates of RNA-seq before treatment and after 20–24 h of ATRA treatment. ATRA inhibits PML::RARA and induces maturation of the promyelocytes into mature blood cells. Differential expression of lincRNAs between the timepoints was obtained using DEseq2 (v1.42.1). Statistical analysis was conducted using one-way ANOVA with Holm–Šídák multiple comparisons correction.

## 5. Conclusions

This study presents LIRA (Long Intergenic Noncoding RNA Annotator), a freely available pipeline for the identification of novel lincRNAs. Using LIRA, we expand the annotated lincRNA landscape in AML by 27%. Comprehensive profiling of 898 AML and healthy samples reveals that, similar to protein-coding genes, lincRNA expression patterns closely reflect established genetic AML subclasses. Weighted gene co-expression network analysis uncovers modules that correlate with these subclasses. Altogether, this work provides a resource for functional studies, facilitating the exploration of lincRNAs as biomarkers and therapeutic targets, with the ultimate goal of improving patient outcomes in AML.

## Data Availability

The TARGET-AML, BEATAML1.0 and TCGA-LAML cohort data are available through https://portal.gdc.cancer.gov/ under controlled access (accessed on 9 February 2022). The AML-05 cohort and PML::RARA ATRA treatment data can be requested from the European Genome Archive (EGA) at EGAD00001005078 and EGAD00001002356, respectively. The remaining data is available from the Gene Expression Omnibus (GEO): RNA-seq data from 6 KMT2A:MLLT3 samples used for validation at GSE79899, ChIP-seq data at GSE89336, CAGE-seq data at GSE204707 and GSE296865, KMT2A::MLLT3 degradation and small molecule inhibition at GSE173574. Pipeline LIRA will be available from GitHub as a Nextflow pipeline (https://github.com/DGilissen/LIRA, accessed on 16 April 2026) upon publication.

## Supplementary Materials

The following supporting information can be downloaded at: https://www.mdpi.com/article/10.3390/ncrna12030018/s1, Figure S1: LincRNAs have fewer exons and are smaller compared to protein-coding genes; Figure S2: UMAP plots of separate cohorts, also showing grouping of genetic AML subclasses; Figure S3: WGCNA modules vary in size and fraction of novel lincRNAs; Table S1: Genomic location (GRCh38) of the 1560 newly identified intergenic long noncoding RNAs; Table S2: batch corrected, non-normalized counts of known and novel lincRNAs for 878 AML and 20 healthy samples (in order of: BEATAML, TCGA-LAML, TARGET-AML, AML-05); Table S3: batch corrected, non-normalized counts of protein coding transcripts for 878 AML and 20 healthy samples (in order of: BEATAML, TCGA-LAML, TARGET-AML, AML-05); Table S4: LincRNAs included in each WGCNA module; Table S5: Correlation (upper) and *p*-value (lower) for each WGCNA module; Table S6: Known lincRNAs in the top 1000 most variable lincRNAs that have been previously associated with leukemia; Table S7: Number of samples in each subclass used for WGCNA age comparison; Table S8: Gene IDs of lincRNAs included in each WGCNA module in the pediatric vs. adult comparison (Figure 5A); Table S9: Correlation (upper) and *p*-value (lower) for each WGCNA module in the pediatric versus adult comparison.

## Abbreviations

The following abbreviations are used in this manuscript:

CAGE	5′ Cap Analysis of Gene Expression
AML	Acute myeloid leukemia
lincRNA	Long intergenic noncoding RNA
LIRA	Long intergenic noncoding RNA annotator
UMAP	Uniform manifold approximation and projection
WGCNA	Weighted gene co-expression network analysis
