# Supplementary material for: Comprehensive lincRNA Transcriptome in Acute Myeloid Leukemia: Integrating Known and Newly Identified lincRNAs Across Pediatric and Adult Cohorts
Source: Noncoding RNA. 2026 May 27;12(3):18. doi: 10.3390/ncrna12030018 (PMC13305162; doi:10.3390/ncrna12030018)
Supplement: Supplementary file 1 [file ncrna-12-00018-s001.zip › supplementary_figures_tables.pdf]

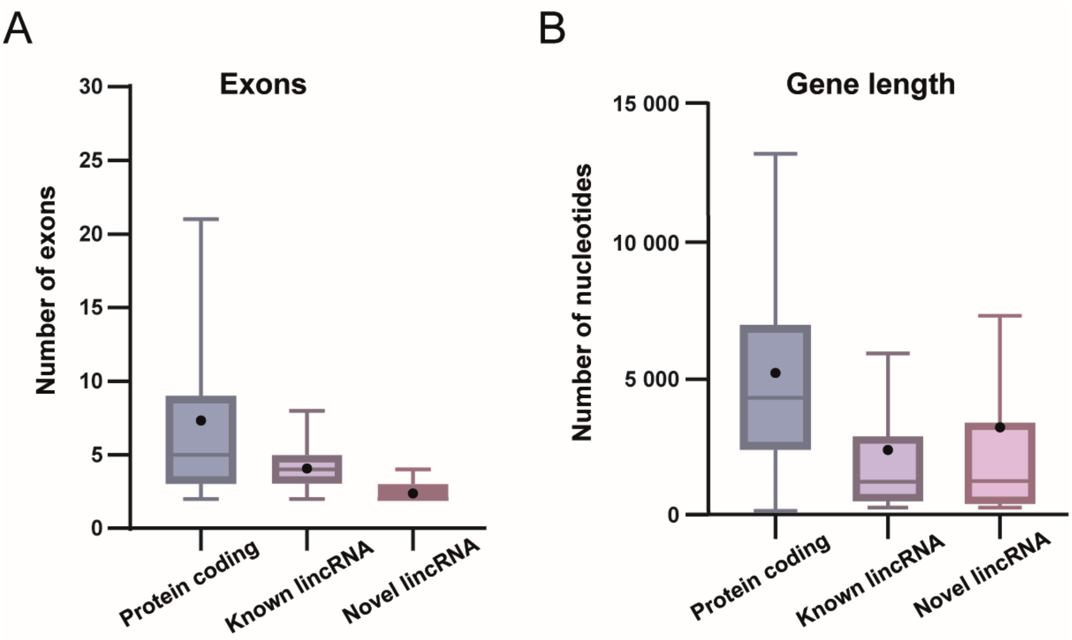

Figure S1. LincRNAs have fewer exons and are smaller compared to protein-coding genes. Size comparison between protein-coding genes (*n*=19963), known lincRNAs (*n*=5858), and novel lincRNAs (*n*=1560) across the 898 analyzed samples. (**A**) shows exon counts per transcript and (**B**) depicts gene length (genomic start of the first transcript to genomic end of the last transcript of the gene). Protein-coding genes had more exons and were longer compared to lincRNAs. Much smaller size differences were observed between known and novel lincRNA. All means (marked by black dots) were significantly different ($p < 0.0001$, one-way ANOVA, Tukey corrected). Graphs were created using GraphPad.

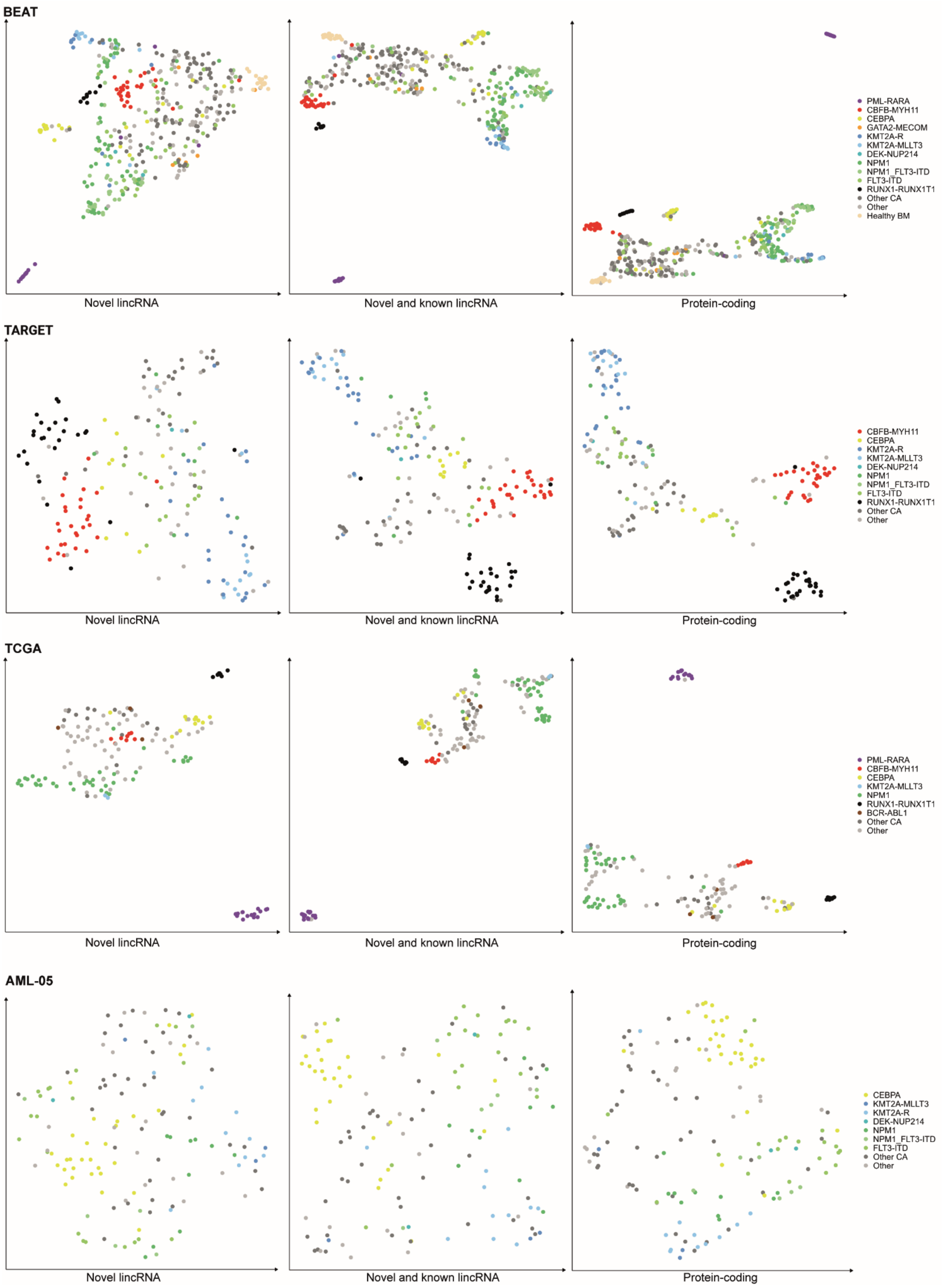

BEAT
Novel lincRNA
Novel and known lincRNA
Protein-coding
PML-RARA
CBFB-MYH11
CEBPA
GATA2-MECOM
KMT2A-R
KMT2A-MLLT3
DEK-NUP214
NPM1
NPM1_FLT3-ITD
FLT3-ITD
RUNX1-RUNX1T1
Other CA
Other
Healthy BM
TARGET
Novel lincRNA
Novel and known lincRNA
Protein-coding
CBFB-MYH11
CEBPA
KMT2A-R
KMT2A-MLLT3
DEK-NUP214
NPM1
NPM1_FLT3-ITD
FLT3-ITD
RUNX1-RUNX1T1
Other CA
Other
TCGA
Novel lincRNA
Novel and known lincRNA
Protein-coding
PML-RARA
CBFB-MYH11
CEBPA
KMT2A-MLLT3
NPM1
RUNX1-RUNX1T1
BCR-ABL1
Other CA
Other
AML-05
Novel lincRNA
Novel and known lincRNA
Protein-coding
CEBPA
KMT2A-MLLT3
KMT2A-R
DEK-NUP214
NPM1
NPM1_FLT3-ITD
FLT3-ITD
Other CA
Other

Figure S2. UMAP plots of separate cohorts also show grouping of genetic AML subclasses. UMAP visualizations showing, from top to bottom, the gene expression of samples in BEATAML1.0 (410 AML patients, 19 healthy bone marrow (BM)), TARGET-AML (178 AML), TCGA-LAML (151 AML) and AML-05 (139 AML) cohorts. From left to right, graphs are based on the top 1000 most variable genes of: novel lincRNAs, combined known and novel lincRNAs, and protein-coding genes. Each point represents a sample, and colors denote genetically defined AML subtypes or healthy BM. Cytogenic aberrations (CA) with low occurrences are labelled Other CA.

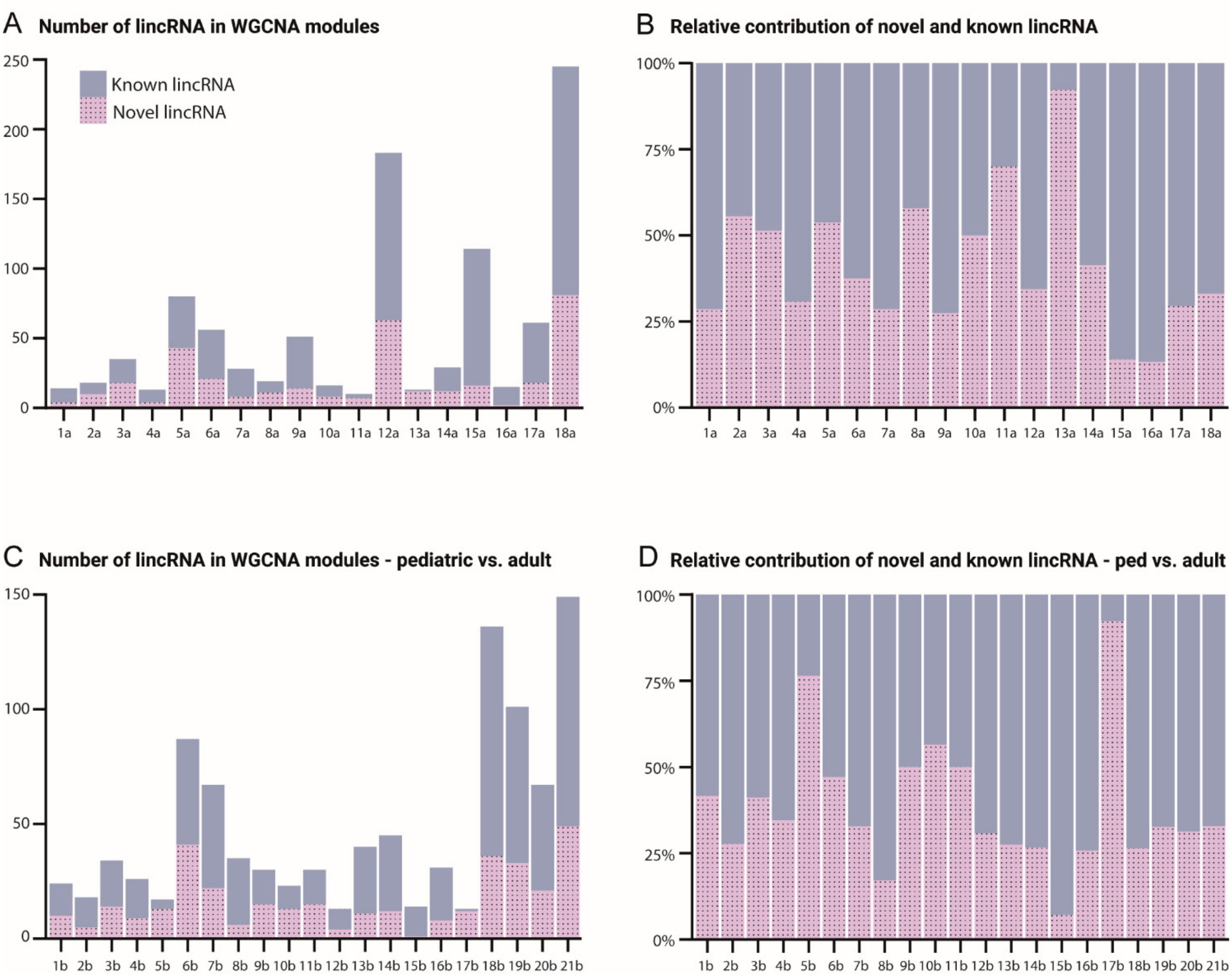

Figure S3. WGCNA modules vary in size and fraction of novel lincRNAs. The number of lincRNA in each WGCNA module (**A**, **C**) and relative contribution of novel and known transcripts per WGCNA module (**B**, **D**) display that the size and makeup differed between modules. The upper figures (**A–B**) show the WGCNA modules using all samples (Figure 3D). The lower figures (**C–D**) show the modules resulting from the comparison between pediatric and adult samples (Figure 5A), in which only *CBFB::MYH11*, *CEBPA* mutated, *FLT3* internal tandem duplication, *KMT2A* rearranged and *RUNX1::RUNX1T1* samples were considered. Both the overall and age-dependent analysis used the same top 1000 most variable lincRNAs. The last module (18a in **A–B**, 21b in **C–D**) contains lincRNA that were not part of any co-expression module.

Table S4. LincRNAs included in each WGCNA module.

Mod.	Size	LincRNA
A1	14	ENSG00000257883.1; ENSG00000257654.1; ENSG00000231106.2; ENSG00000232229.6; ENSG00000280560.2; LIRA.1363; LIRA.628; ENSG00000232591.2; LIRA.1198; LIRA.937; ENSG00000229484.1; ENSG00000237166.2; ENSG00000285867.1; ENSG00000228157.4
A2	18	ENSG00000234177.5; LIRA.1062; ENSG00000238266.2; LIRA.1111; LIRA.888; LIRA.1110; ENSG00000233410.1; LIRA.1291; LIRA.695; LIRA.321; ENSG00000250739.1; LIRA.819; LIRA.893; ENSG00000235492.1; LIRA.894; ENSG00000259594.6; ENSG00000283072.3; ENSG00000260645.2
A3	35	ENSG00000251381.8; ENSG00000249297.2; LIRA.332; LIRA.1028; LIRA.1057; LIRA.803; ENSG00000278090.3; ENSG00000258018.2; LIRA.143; LIRA.236; ENSG00000254101.7; LIRA.1058; ENSG00000248703.2; ENSG00000251584.2; LIRA.102; LIRA.697; LIRA.806; ENSG00000267337.2; ENSG00000249464.6; ENSG00000249926.2; ENSG00000228221.6; LIRA.762; ENSG00000230400.3; ENSG00000239482.6; LIRA.967; LIRA.763; LIRA.1080; LIRA.81; LIRA.310; ENSG00000256995.8; LIRA.535; LIRA.1009; ENSG00000233208.6; ENSG00000250266.2; ENSG00000235021.1
A4	13	ENSG00000204603.7; ENSG00000237361.3; LIRA.324; ENSG00000284237.1; ENSG00000230731.3; ENSG00000234928.1; ENSG00000231566.2; LIRA.322; ENSG00000178248.11; ENSG00000189275.4; ENSG00000254343.2; LIRA.253; LIRA.117
A5	80	ENSG00000224739.2; ENSG00000230690.2; LIRA.481; LIRA.707; ENSG00000224400.5; ENSG00000259042.3; ENSG00000285354.1; LIRA.31; ENSG00000251273.4; LIRA.33; LIRA.175; ENSG00000244620.1; ENSG00000232555.2; LIRA.188; LIRA.484; LIRA.480; ENSG00000228876.4; ENSG00000232444.1; LIRA.1183; LIRA.30; LIRA.1188; ENSG00000226777.7; LIRA.1482; LIRA.29; ENSG00000258084.6; ENSG00000231131.8; LIRA.38; LIRA.946; ENSG00000253619.1; LIRA.986; ENSG00000224099.2; LIRA.1243; ENSG00000250072.6; LIRA.1261; LIRA.166; LIRA.104; ENSG00000260876.6; LIRA.748; LIRA.983; ENSG00000225285.1; LIRA.250; LIRA.726; LIRA.1347; LIRA.163; ENSG00000271893.1; LIRA.1471; ENSG00000231829.4; ENSG00000234962.6; LIRA.1371; ENSG00000198685.4; ENSG00000255418.6; ENSG00000223466.2; LIRA.939; LIRA.223; ENSG00000251002.8; ENSG00000226581.2; ENSG00000248690.8; ENSG00000274317.2; LIRA.880; LIRA.839; ENSG00000242258.1; LIRA.1106; LIRA.1150; ENSG00000232837.1; ENSG00000230105.1; LIRA.1285; LIRA.216; ENSG00000212939.2; LIRA.1054; ENSG00000233834.6; LIRA.497; ENSG00000261390.6; ENSG00000250634.6; LIRA.1218; LIRA.282; ENSG00000285571.1; ENSG00000223985.2; LIRA.498; LIRA.1148; LIRA.494
A6	56	ENSG00000253474.2; ENSG00000229425.3; ENSG00000233236.1; LIRA.698; LIRA.700; ENSG00000227674.3; LIRA.330; LIRA.97; LIRA.222; ENSG00000166770.11; LIRA.366; ENSG00000214145.7; ENSG00000242268.3; ENSG00000260455.2; ENSG00000260409.1; LIRA.576; LIRA.1170; ENSG00000253522.6; ENSG00000231533.2; ENSG00000215386.13; ENSG00000272168.8; ENSG00000280623.1; LIRA.833; ENSG00000258710.8; ENSG00000254396.1; LIRA.1187; ENSG00000225930.4; LIRA.1184; ENSG00000249258.2; LIRA.1152; LIRA.1138; ENSG00000237484.6; LIRA.280; LIRA.1319; ENSG00000266604.1; ENSG00000228484.3; ENSG00000226686.8; ENSG00000225948.2; LIRA.337; LIRA.92; ENSG00000267767.3; ENSG00000257239.1; LIRA.1293; LIRA.1022; ENSG00000234283.1; ENSG00000254416.6; ENSG00000230499.1; LIRA.543; ENSG00000255250.2; ENSG00000231439.4; ENSG00000259072.2; ENSG00000227017.1; ENSG00000262786.1; ENSG00000270071.3; LIRA.89; ENSG00000225742.6;
A7	28	ENSG00000224968.2; LIRA.378; ENSG00000259439.2; LIRA.74; LIRA.1169; ENSG00000263958.2; ENSG00000285662.2; ENSG00000237667.7; ENSG00000223930.7; LIRA.1265; ENSG00000255248.9; ENSG00000264404.3; ENSG00000225493.1; ENSG00000245694.10; ENSG00000249618.6; ENSG00000236502.1; ENSG00000284240.1; LIRA.391; ENSG00000225156.2; LIRA.244; ENSG00000223751.1; ENSG00000273415.3; LIRA.1197; LIRA.376; ENSG00000259725.1; ENSG00000204241.8; ENSG00000227579.6; ENSG00000259711.1
A8	19	LIRA.1069; ENSG00000265369.3; LIRA.1068; LIRA.1072; ENSG00000263677.2; LIRA.1067; ENSG00000225087.2; LIRA.1073; LIRA.291; LIRA.315; ENSG00000235578.1; LIRA.1061; LIRA.1101; ENSG00000223561.7; LIRA.743; ENSG00000230131.6; ENSG00000267130.1; LIRA.616; ENSG00000238078.1
A9	51	ENSG00000257582.5; ENSG00000254006.5; ENSG00000228778.2; ENSG00000227502.3; ENSG00000249797.2; ENSG00000226562.4; ENSG00000229855.9; ENSG00000226673.2; ENSG00000280916.2; ENSG00000122548.5; ENSG00000235244.4; LIRA.1373; ENSG00000253508.1; ENSG00000228065.11; LIRA.945; ENSG00000257114.3; LIRA.1314; ENSG00000262877.5; ENSG00000224810.1; ENSG00000233922.3; ENSG00000268240.1; ENSG00000232046.7; LIRA.1417; LIRA.954; ENSG00000250891.2; LIRA.115; LIRA.960; LIRA.952; LIRA.955; LIRA.405; ENSG00000175772.11; ENSG00000281091.3; ENSG00000214870.9; LIRA.970; LIRA.404; ENSG00000204588.5; ENSG00000233760.2; ENSG00000224687.2; ENSG00000262585.1; ENSG00000274605.2; LIRA.341; ENSG00000235888.3; ENSG00000262188.2; ENSG00000231527.7; ENSG00000259129.6;

		ENSG00000233639.7; ENSG00000233008.6; ENSG00000232079.7; ENSG00000258711.2; LIRA.1374; ENSG00000264451.1
A10	16	LIRA.1502; LIRA.435; LIRA.338; ENSG00000265728.1; LIRA.112; LIRA.113; ENSG00000272808.4; ENSG00000272692.2; LIRA.1428; ENSG00000231711.2; ENSG00000267247.1; LIRA.1397; ENSG00000231010.1; ENSG00000229557.1; ENSG00000232386.9; LIRA.1031
A11	10	LIRA.403; ENSG00000273443.1; LIRA.407; LIRA.1214; LIRA.1139; ENSG00000233721.1; ENSG00000253227.2; LIRA.1100; LIRA.368; LIRA.1362
A12	183	LIRA.195; ENSG00000246100.4; ENSG00000185168.5; ENSG00000248810.2; ENSG00000231412.2; ENSG00000261222.3; LIRA.659; ENSG00000231680.1; LIRA.1040; ENSG00000234426.3; ENSG00000228742.11; ENSG00000224397.7; LIRA.202; ENSG00000176320.2; LIRA.1238; ENSG00000281162.2; LIRA.316; ENSG00000271952.2; ENSG00000136315.4; ENSG00000228058.2; ENSG00000197503.4; LIRA.319; ENSG00000232063.2; ENSG00000261172.1; LIRA.318; ENSG00000254952.1; ENSG00000270087.5; ENSG00000227066.2; LIRA.1454; LIRA.186; ENSG00000282572.2; LIRA.1075; LIRA.1146; LIRA.1324; ENSG00000204960.7; LIRA.796; ENSG00000230138.2; ENSG00000285492.1; ENSG00000258831.1; LIRA.346; ENSG00000265519.1; ENSG00000224307.2; LIRA.637; ENSG00000234506.5; ENSG00000246430.7; ENSG00000255801.1; LIRA.1133; ENSG00000227508.6; LIRA.636; ENSG00000285163.1; ENSG00000238042.5; ENSG00000284930.1; ENSG00000262097.2; ENSG00000213373.7; LIRA.43; ENSG00000249173.6; ENSG00000267364.2; LIRA.272; ENSG00000269877.3; ENSG00000230836.1; ENSG00000255666.7; LIRA.1242; LIRA.5; ENSG00000253821.2; LIRA.1143; LIRA.875; LIRA.630; ENSG00000265743.1; ENSG00000235478.5; ENSG00000228022.6; ENSG00000229792.1; ENSG00000285040.1; ENSG00000226476.6; ENSG00000233746.2; ENSG00000180769.10; LIRA.765; ENSG00000285954.1; ENSG00000214797.3; LIRA.1461; ENSG00000249771.2; ENSG00000269667.2; LIRA.878; ENSG00000222004.7; ENSG00000203999.9; LIRA.1134; ENSG00000226453.2; LIRA.1231; LIRA.1333; ENSG00000254639.1; ENSG00000248455.6; LIRA.563; LIRA.655; ENSG00000227925.2; ENSG00000205300.3; ENSG00000254813.5; LIRA.1123; LIRA.593; LIRA.760; LIRA.191; ENSG00000236403.2; LIRA.1342; ENSG00000232053.7; LIRA.1052; LIRA.947; ENSG00000253214.2; LIRA.858; ENSG00000230107.1; LIRA.565; ENSG00000232721.2; ENSG00000250274.2; LIRA.62; ENSG00000278214.1; ENSG00000225127.3; ENSG00000255363.3; ENSG00000237797.1; ENSG00000229771.2; ENSG00000258867.6; ENSG00000285630.1; ENSG00000237879.1; ENSG00000237476.1; ENSG00000236914.4; LIRA.612; ENSG00000227082.3; LIRA.1097; LIRA.701; ENSG00000179082.3; LIRA.1378; ENSG00000237525.7; LIRA.453; ENSG00000253507.5; LIRA.392; ENSG00000237803.6; ENSG00000260430.2; ENSG00000196758.3; ENSG00000230649.3; ENSG00000259717.1; ENSG00000223914.4; LIRA.632; ENSG00000251230.6; ENSG00000229981.8; LIRA.1145; ENSG00000277851.2; ENSG00000267711.1; ENSG00000232316.2; LIRA.1114; LIRA.1418; ENSG00000257595.3; LIRA.384; ENSG00000231528.3; ENSG00000258754.8; ENSG00000259130.2; LIRA.549; ENSG00000253123.4; LIRA.666; ENSG00000226004.1; ENSG00000226644.5; ENSG00000253177.2; ENSG00000229891.2; LIRA.606; ENSG00000267123.7; LIRA.229; ENSG00000254288.1; ENSG00000255557.1; ENSG00000255491.3; LIRA.73; ENSG00000225684.4; ENSG00000235770.6; LIRA.488; LIRA.971; ENSG00000260507.1; ENSG00000229454.1; ENSG00000254802.1; ENSG00000226091.7; ENSG00000267659.5; ENSG00000253161.5; ENSG00000259269.2; ENSG00000232539.1; ENSG00000258476.6; ENSG00000260528.5; ENSG00000256955.2; ENSG00000241280.1; LIRA.657; LIRA.1131
A13	13	LIRA.679; LIRA.670; LIRA.678; LIRA.675; LIRA.676; LIRA.668; LIRA.680; LIRA.667; LIRA.691; LIRA.683; ENSG00000237807.4; LIRA.669; LIRA.684
A14	29	LIRA.909; ENSG00000283646.2; ENSG00000283445.1; LIRA.182; ENSG00000251127.2; LIRA.1030; ENSG00000259070.7; LIRA.1447; LIRA.278; LIRA.181; LIRA.120; ENSG00000258919.1; ENSG00000258819.2; ENSG00000253315.1; LIRA.987; ENSG00000232680.2; LIRA.1341; ENSG00000254872.3; ENSG00000233755.1; LIRA.151; ENSG00000230623.6; ENSG00000285639.1; LIRA.648; LIRA.121; ENSG00000258404.1; ENSG00000275630.1; ENSG00000259225.7; ENSG00000254789.3; ENSG00000233214.1
A15	114	ENSG00000251129.2; ENSG00000283633.1; LIRA.124; LIRA.651; ENSG00000285578.1; ENSG00000276476.3; ENSG00000235532.1; ENSG00000245812.3; ENSG00000231419.6; ENSG00000253967.1; ENSG00000261804.2; ENSG00000255652.4; ENSG00000234170.6; ENSG00000257647.1; ENSG00000267568.6; ENSG00000280953.2; LIRA.615; ENSG00000229205.5; ENSG00000250125.4; ENSG00000285079.1; LIRA.125; ENSG00000267521.1; ENSG00000256001.2; ENSG00000228495.2; ENSG00000132204.14; ENSG00000267339.7; ENSG00000248360.7; ENSG00000267325.2; ENSG00000228709.1; ENSG00000234184.6; ENSG00000226383.7; LIRA.1446; ENSG00000251152.1; ENSG00000244649.5; ENSG00000205056.8; ENSG00000187621.15; ENSG00000254266.6; LIRA.613; ENSG00000226423.1; ENSG00000254166.3; ENSG00000226530.2; ENSG00000260302.3; ENSG00000245954.8; ENSG00000249574.1; ENSG00000266088.6; ENSG00000261645.7; ENSG00000273297.2; ENSG00000253364.2; ENSG00000268621.6; ENSG00000285847.2; ENSG00000235621.9; ENSG00000246223.9; ENSG00000259153.2; ENSG00000256128.6; ENSG00000270816.6; ENSG00000249667.1; ENSG00000247970.2;

		LIRA.254; ENSG00000224957.6; LIRA.802; ENSG00000189238.6; LIRA.136; ENSG00000228590.2; LIRA.647; ENSG00000248991.1; ENSG00000283141.1; ENSG00000237372.4; ENSG00000267414.1; ENSG00000235726.7; ENSG00000246084.5; ENSG00000231682.1; ENSG00000258534.1; ENSG00000235665.6; ENSG00000230317.2; ENSG00000263667.3; ENSG00000224137.1; ENSG00000232884.9; ENSG00000227920.3; LIRA.915; ENSG00000250415.1; ENSG00000224610.1; ENSG00000249395.4; ENSG00000242147.3; ENSG00000285535.2; ENSG00000254275.6; ENSG00000197880.9; ENSG00000233828.5; ENSG00000267501.1; LIRA.397; ENSG00000205628.4; ENSG00000256427.2; ENSG00000222032.1; ENSG00000249550.7; ENSG00000226856.7; LIRA.243; ENSG00000256424.2; ENSG00000235576.1; ENSG00000204110.6; ENSG00000284624.1; LIRA.1021; LIRA.345; ENSG00000225579.2; ENSG00000285517.1; ENSG00000233191.2; ENSG00000226320.6; ENSG00000245164.8; LIRA.795; ENSG00000250390.2; ENSG00000229628.1; ENSG00000241316.8; ENSG00000241657.1; ENSG00000225978.3; ENSG00000258702.3; ENSG00000267243.6
A16	15	ENSG00000268555.2; ENSG00000267506.5; ENSG00000267466.1; ENSG00000228824.7; ENSG00000241743.4; LIRA.830; ENSG00000251443.2; LIRA.177; ENSG00000228215.3; ENSG00000248373.6; ENSG00000243701.7; ENSG00000281852.1; ENSG00000257660.5; ENSG00000259705.1; ENSG00000204277.1
A17	61	ENSG00000214548.18; ENSG00000271736.2; ENSG00000249790.3; LIRA.1194; LIRA.1192; ENSG00000285933.1; ENSG00000250334.6; ENSG00000224177.8; ENSG00000232265.8; ENSG00000224259.7; LIRA.211; LIRA.1299; ENSG00000253154.2; ENSG00000258114.1; LIRA.63; LIRA.48; ENSG00000250501.3; ENSG00000225746.13; ENSG00000251434.1; ENSG00000233237.8; LIRA.262; ENSG00000267279.2; LIRA.1403; LIRA.239; LIRA.985; ENSG00000253394.6; ENSG00000253819.2; LIRA.1019; ENSG00000277526.5; ENSG00000214049.8; ENSG00000185904.12; ENSG00000253986.1; LIRA.800; LIRA.141; LIRA.1409; ENSG00000285486.1; ENSG00000285557.1; ENSG00000225807.2; ENSG00000254972.1; ENSG00000236849.6; LIRA.929; LIRA.327; ENSG00000255364.1; ENSG00000231246.2; LIRA.493; ENSG00000235142.10; ENSG00000249406.3; ENSG00000254810.1; ENSG00000224184.6; ENSG00000244128.7; ENSG00000228541.1; LIRA.1089; ENSG00000234261.4; ENSG00000255325.2; ENSG00000250101.1; ENSG00000233593.10; ENSG00000253369.2; ENSG00000175746.6; ENSG00000227733.10; ENSG00000282142.2; ENSG00000224893.7
A18 (rest)	245	ENSG00000229807.13; ENSG00000176728.10; ENSG00000231535.7; ENSG00000225231.2; ENSG00000204792.2; ENSG00000229236.3; ENSG00000253853.3; ENSG00000251209.9; ENSG00000206195.11; ENSG00000227706.4; LIRA.541; LIRA.1088; ENSG00000223749.11; ENSG00000285534.1; ENSG00000234147.2; ENSG00000230426.4; ENSG00000250986.1; ENSG00000205611.5; ENSG00000226900.2; ENSG00000247134.7; ENSG00000189229.11; ENSG00000283235.2; LIRA.768; ENSG00000247516.8; ENSG00000248874.5; LIRA.371; ENSG00000254319.6; ENSG00000227403.2; LIRA.1498; ENSG00000230387.4; ENSG00000250337.7; ENSG00000239572.3; LIRA.1557; LIRA.396; LIRA.1552; ENSG00000247765.2; ENSG00000130600.19; ENSG00000258498.8; LIRA.1055; ENSG00000177335.11; LIRA.1432 ENSG00000266976.3; ENSG00000271584.2; LIRA.706; ENSG00000225172.6; ENSG00000234665.9; ENSG00000267374.2; LIRA.837; ENSG00000256209.2; LIRA.538; ENSG00000275216.2; ENSG00000236209.1; ENSG00000253140.2; LIRA.1260; ENSG00000226051.8; ENSG00000283183.2; LIRA.592; LIRA.930; ENSG00000236834.1; ENSG00000239268.3; ENSG00000285722.1; ENSG00000275232.1; LIRA.1233; LIRA.1413; LIRA.394; ENSG00000251555.1; ENSG00000234690.7; LIRA.1507; ENSG00000253764.3; LIRA.1558; LIRA.1091; ENSG00000230631.1; ENSG00000277128.2; ENSG00000240350.3; ENSG00000265179.7; ENSG00000232310.8; ENSG00000205837.7; ENSG00000263745.7; ENSG00000243144.7; LIRA.745; ENSG00000255980.1; LIRA.255; ENSG00000272438.1; ENSG00000244706.4; LIRA.1318; LIRA.1065; ENSG00000285108.1; ENSG00000253217.1; LIRA.256; ENSG00000283458.1; LIRA.746; ENSG00000227060.7; LIRA.1076; LIRA.580; LIRA.1039; ENSG00000231476.1; ENSG00000205293.5; ENSG00000230699.2; ENSG00000237531.6; LIRA.386; ENSG00000283573.1; ENSG00000269994.3; ENSG00000239467.6; ENSG00000230490.3; ENSG00000224559.2; LIRA.1222; ENSG00000235994.4; ENSG00000249159.6; LIRA.1162; ENSG00000234944.1; LIRA.788; ENSG00000230628.1; ENSG00000251138.7; LIRA.457; ENSG00000267780.2; ENSG00000237471.2; ENSG00000236700.6; ENSG00000223486.1; ENSG00000272763.1; ENSG00000253301.6; LIRA.240; LIRA.1275; LIRA.317; ENSG00000250241.6; ENSG00000223764.2; ENSG00000259692.6; ENSG00000227467.3; ENSG00000246363.3; ENSG00000223403.6; ENSG00000255571.9; LIRA.224; LIRA.744; LIRA.935; LIRA.1025; ENSG00000230876.8; ENSG00000227107.1; ENSG00000234464.2; LIRA.889; LIRA.738; LIRA.375; ENSG00000256982.2; LIRA.483; LIRA.41; ENSG00000229660.1; ENSG00000231877.1; ENSG00000253230.9; ENSG00000273102.1; ENSG00000250102.6; ENSG00000260289.2; LIRA.825; LIRA.1189; ENSG00000226954.1; LIRA.1312; LIRA.363; ENSG00000281202.2; LIRA.730; ENSG00000258517.1; ENSG00000237346.2; LIRA.1438; ENSG00000276850.5; LIRA.764; LIRA.283; ENSG00000231081.1; ENSG00000231453.1; LIRA.618; ENSG00000260664.2; ENSG00000285624.2; LIRA.1102; ENSG00000264695.1; ENSG00000234622.6; ENSG00000267924.1; ENSG00000256783.1; ENSG00000235151.1; LIRA.847; ENSG00000215808.4; ENSG00000282418.1; ENSG00000273172.1; LIRA.579; ENSG00000223855.2; LIRA.1353; ENSG00000261327.5; ENSG00000237357.2; ENSG00000230563.4; ENSG00000223342.2; ENSG00000250786.2;

	LIRA.1491; ENSG00000223511.7; ENSG00000226397.8; ENSG00000250658.1; LIRA.10; ENSG00000234350.5; ENSG00000283422.1; ENSG00000256546.2; LIRA.260; ENSG00000239941.1; ENSG00000233081.1; ENSG00000240405.7; ENSG00000238107.1; LIRA.87; ENSG00000232190.3; ENSG00000223387.6; ENSG00000249937.8; ENSG00000259420.5; LIRA.251; ENSG00000285838.2; ENSG00000226519.2; LIRA.842; LIRA.812; LIRA.1346; ENSG00000248187.1; ENSG00000225489.7; ENSG00000237422.2; ENSG00000232977.7; LIRA.883; LIRA.209; ENSG00000259664.3; ENSG00000226237.2; ENSG00000256124.6; ENSG00000224652.2; ENSG00000227888.4; ENSG00000271201.1; LIRA.571; ENSG00000259218.6; ENSG00000229308.1; ENSG00000253449.1; ENSG00000267372.2; ENSG00000223806.8; LIRA.1066; LIRA.259; LIRA.196; ENSG00000227683.1; ENSG00000250041.3; LIRA.150; LIRA.402; LIRA.201; ENSG00000228139.1; LIRA.1180; ENSG00000284430.1; LIRA.1503; ENSG00000231826.6; ENSG00000242516.2; ENSG00000233405.2; ENSG00000253395.1; LIRA.1295; ENSG00000249241.1

Gene IDs of lincRNAs included in each WGCNA module (Figure 3D), with novel lincRNA denoted LIRA.XXX..

Table S5. Correlation (upper) and p-value (lower) for each WGCNA module.

	PML::RARA	CBFB::MYH11	CEBPA	GATA2::MECOM	KMT2A-R	DEK::NUP214	NPM1	FLT3-ITD	RUNX1::RUNX1T1	BCR::ABL1	Other CA	Other	Healthy BM
A1	-0.1949	0.2828	0.1897	0.0039	0.1226	-0.0214	-0.3705	-0.0331	0.1339	-0.0093	-0.0263	0.0252	0.0192
A2	0.3394	0.0456	0.4499	-0.0245	-0.1154	0.0007	-0.1957	-0.0495	0.0748	-0.0459	-0.1081	-0.0887	-0.0755
A3	-0.1402	0.2250	0.1188	0.0479	-0.1936	-0.0252	-0.4129	-0.0347	0.2962	0.0335	0.1747	0.0548	-0.0070
A4	-0.0451	0.3056	0.1021	0.0437	-0.2587	0.0464	-0.2116	0.0462	0.2375	-0.0330	0.0265	-0.0184	-0.1150
A5	0.0031	-0.1076	0.2033	0.0712	-0.2613	0.0886	0.1170	0.1330	-0.1206	-0.0371	-0.0232	-0.0102	-0.0658
A6	-0.1522	0.0076	0.0703	0.1535	-0.2471	0.0025	-0.1651	0.0272	-0.0069	0.0540	0.2072	0.0900	-0.0131
A7	0.6414	-0.1621	-0.0776	-0.0320	0.0113	0.0359	0.1526	-0.0446	-0.0733	-0.0292	-0.0846	-0.1234	-0.0438
A8	0.0703	-0.1235	0.0861	-0.0237	0.3116	0.0744	0.0896	0.0432	-0.0871	-0.0656	-0.1867	-0.0952	-0.0457
A9	-0.1370	-0.3141	-0.2267	0.0066	0.0900	0.1051	0.4983	0.1110	-0.2697	-0.0129	0.0033	-0.0640	-0.0324
A10	-0.2185	-0.2327	-0.2954	0.0014	0.3729	0.0968	0.4398	0.0714	-0.1314	-0.0300	-0.0875	-0.1015	-0.0855
A11	-0.0193	-0.1077	-0.1272	-0.0422	0.0676	-0.0150	0.1274	-0.0170	-0.0653	-0.0099	0.1044	-0.0190	-0.0821
A12	-0.1319	0.0842	-0.1534	-0.0260	0.2752	-0.0114	-0.0004	-0.1133	-0.1093	-0.0041	0.0118	0.0079	0.1251
A13	-0.0633	-0.0258	0.0054	-0.0423	-0.0274	-0.0316	-0.0839	-0.0926	0.0160	-0.0226	0.0524	0.0802	0.2259
A14	-0.0984	-0.1081	-0.0494	0.0141	-0.0942	-0.0094	-0.1298	-0.0924	-0.0235	0.0118	0.2381	0.1207	0.1444
A15	-0.0607	-0.1227	-0.1140	0.0461	0.0266	-0.0001	-0.1905	-0.1662	-0.0347	0.0340	0.1784	0.1469	0.3887
A16	-0.1556	-0.2581	-0.0949	0.1618	-0.0504	-0.0061	0.0013	-0.0416	-0.0052	0.0693	0.1869	0.0982	0.1138
A17	-0.0668	-0.2493	0.0629	0.0647	-0.0616	-0.0298	-0.1499	-0.1112	-0.1147	0.0540	0.2018	0.1382	0.3175
A18	0.0042	-0.1631	-0.0544	0.0108	0.3089	0.0446	-0.1084	-0.1453	0.0298	0.0321	0.0690	-0.0017	0.0814

	PML::RARA	CBFB::MYH11	CEBPA	GATA2::MECOM	KMT2A-R	DEK::NUP214	NPM1	FLT3-ITD	RUNX1::RUNX1T1	BCR::ABL1	Other CA	Other	Healthy BM
A1	3.9E-09	5.8E-18	1.0E-08	9.1E-01	2.3E-04	5.2E-01	1.4E-30	3.2E-01	5.7E-05	7.8E-01	4.3E-01	4.5E-01	5.7E-01
A2	1.3E-25	1.7E-01	6.7E-46	4.6E-01	5.3E-04	9.8E-01	3.4E-09	1.4E-01	2.5E-02	1.7E-01	1.2E-03	7.8E-03	2.4E-02
A3	2.5E-05	9.3E-12	3.6E-04	1.5E-01	5.0E-09	4.5E-01	3.1E-38	3.0E-01	1.3E-19	3.2E-01	1.4E-07	1.0E-01	8.3E-01
A4	1.8E-01	7.7E-21	2.2E-03	1.9E-01	3.5E-15	1.7E-01	1.5E-10	1.7E-01	5.8E-13	3.2E-01	4.3E-01	5.8E-01	5.6E-04
A5	9.3E-01	1.3E-03	8.0E-10	3.3E-02	1.8E-15	7.9E-03	4.5E-04	6.5E-05	2.9E-04	2.7E-01	4.9E-01	7.6E-01	4.9E-02
A6	4.7E-06	8.2E-01	3.5E-02	3.9E-06	6.1E-14	9.4E-01	6.6E-07	4.2E-01	8.4E-01	1.1E-01	3.7E-10	7.0E-03	7.0E-01
A7	4.0E-105	1.1E-06	2.0E-02	3.4E-01	7.4E-01	2.8E-01	4.4E-06	1.8E-01	2.8E-02	3.8E-01	1.1E-02	2.1E-04	1.9E-01
A8	3.5E-02	2.1E-04	9.9E-03	4.8E-01	1.2E-21	2.6E-02	7.2E-03	2.0E-01	9.1E-03	5.0E-02	1.8E-08	4.3E-03	1.7E-01
A9	3.8E-05	5.5E-22	6.4E-12	8.4E-01	7.0E-03	1.6E-03	1.9E-57	8.7E-04	2.1E-16	7.0E-01	9.2E-01	5.5E-02	3.3E-01
A10	3.7E-11	1.7E-12	1.6E-19	9.7E-01	5.7E-31	3.7E-03	9.9E-44	3.3E-02	7.9E-05	3.7E-01	8.7E-03	2.3E-03	1.0E-02
A11	5.6E-01	1.2E-03	1.3E-04	2.1E-01	4.3E-02	6.5E-01	1.3E-04	6.1E-01	5.1E-02	7.7E-01	1.7E-03	5.7E-01	1.4E-02
A12	7.4E-05	1.2E-02	3.9E-06	4.4E-01	4.7E-17	7.3E-01	9.9E-01	6.8E-04	1.0E-03	9.0E-01	7.2E-01	8.1E-01	1.7E-04
A13	5.8E-02	4.4E-01	8.7E-01	2.1E-01	4.1E-01	3.5E-01	1.2E-02	5.5E-03	6.3E-01	5.0E-01	1.2E-01	1.6E-02	7.7E-12
A14	3.2E-03	1.2E-03	1.4E-01	6.7E-01	4.7E-03	7.8E-01	9.7E-05	5.6E-03	4.8E-01	7.2E-01	5.0E-13	2.9E-04	1.4E-05
A15	6.9E-02	2.3E-04	6.2E-04	1.7E-01	4.3E-01	1.0E+00	8.9E-09	5.6E-07	3.0E-01	3.1E-01	7.5E-08	9.9E-06	1.0E-33
A16	2.8E-06	4.0E-15	4.5E-03	1.1E-06	1.3E-01	8.6E-01	9.7E-01	2.1E-01	8.8E-01	3.8E-02	1.7E-08	3.2E-03	6.4E-04
A17	4.6E-02	3.5E-14	6.0E-02	5.3E-02	6.5E-02	3.7E-01	6.5E-06	8.5E-04	5.8E-04	1.1E-01	1.1E-09	3.3E-05	1.9E-22
A18	9.0E-01	9.0E-07	1.0E-01	7.5E-01	2.8E-21	1.8E-01	1.1E-03	1.2E-05	3.7E-01	3.4E-01	3.9E-02	9.6E-01	1.5E-02

P-values above 0.01 are shown grey.

Table S6. Known lincRNA in the top 1000 most variable lincRNA that have been previously associated with leukemia.

LincRNA	Module (Fig. 3D)	Associated AML subtype (if mentioned)	Reference
LINC00865	A1		(1)
LINC01262	A2		(2)
LUNAR1	A3		(3)
LINC00958	A3		(4)
LINC01257	A4	RUNX1::RUNX1T1	(5)
FAM30A	A5		(6)
LINC01770	A5		(7)
LINC01700	A5		(8)
ZNF667-AS1	A6		(9, 10)
PCAT14	A6		(11)
NBAT1	A6		(12)
CASC15	A6	RUNX1::RUNX1T1	(12)
CRNDE	A7	PML::RARA	(9, 10, 12, 13)
LINC02465	A7		(14)
PCAT18	A8	NPM1	(11, 15)
LINC00648	A9		(11)
LINC01979	A9	NPM1	(1, 16)
LINC01978	A9	NPM1	(1)
LINC00899	A10		(17, 18)
LINC01270	A12		(19)
LINC02978	A12	PML::RARA	(10)
RNASE2CP	A12	M0 (undifferentiated AML)	(10)
BLACE	A12		(20)
LINC00470	A15		(21)
LINC00504	A15		(22)
LINC00221	A15		(23, 24)
LINC00861	A15		(10)
DUBR	A16		(25, 26)
MEG3	A17		(9, 10, 25, 27-29)
UCA1	A17	CEBPA	(1, 3, 9, 25, 30)
LINC00839	A17		(31)
DUXAP8	A18		(32)

Table S7. Number of samples in each subclass used for WGCNA age comparison.

Category	Nr. of pediatric samples	Nr. of adult samples	Total nr. of samples
CBFB::MYH11	28	35	63
CEBPA	39	34	73
KMT2A-R#	49	25	74
FLT3-ITD	35	35	70
RUNX1::RUNX1T1	25	16	41
#partners of KMT2A			
KMT2A::MLLT3	15	14	29
KMT2A::MLLT10	12	6	18
KMT2A::ELL	7	2	9
KMT2A::MLLT4	5	1	6
KMT2A::SEPT6	2	0	2
KMT2A::MLLT5	0	2	2
KMT2A::MLLT2	1	0	1
KMT2A::MLLT1	1	0	1
KMT2A other partner	6	0	6

Table S8. Gene IDs of lincRNAs included in each WGCNA module in the pediatric vs. adult comparison (Figure 5A).

Mod.	Size	LincRNA
B1	24	ENSG00000250337.7; LIRA.651; ENSG00000275216.2; LIRA.1169; ENSG00000237667.7; ENSG00000132204.14; ENSG00000254166.3; ENSG00000244649.5; LIRA.1162; ENSG00000255248.9; LIRA.926; ENSG00000230876.8; LIRA.763; LIRA.945; LIRA.74; ENSG00000237807.4; ENSG00000272763.1; LIRA.762; ENSG00000205628.4; ENSG00000268621.6; ENSG00000235726.7; ENSG00000253394.6; LIRA.327; LIRA.457
B2	18	ENSG00000268555.2; ENSG00000229425.3; ENSG00000280916.2; ENSG00000251273.4; ENSG00000250072.6; LIRA.104; LIRA.97; ENSG00000214145.7; ENSG00000268240.1; ENSG00000242268.3; ENSG00000225285.1; LIRA.1187; ENSG00000232837.1; LIRA.92; ENSG00000229017.7; LIRA.833; ENSG00000262188.2; ENSG00000258711.2
B3	34	ENSG00000254006.5; ENSG00000257582.5; ENSG00000228778.2; ENSG00000226562.4; ENSG00000224968.2; ENSG00000226673.2; ENSG00000257114.3; ENSG00000265728.1; ENSG00000262877.5; ENSG00000253764.3; ENSG00000253154.2; LIRA.954; LIRA.1061; LIRA.1065; ENSG00000281091.3; ENSG00000224810.1; LIRA.955; LIRA.404; ENSG00000267247.1; LIRA.1101; LIRA.1054; LIRA.1374; ENSG00000249618.6; ENSG00000231527.7; LIRA.952; ENSG00000233008.6; LIRA.405; LIRA.970; LIRA.244; ENSG00000240405.7; LIRA.1373; ENSG00000238217.6; ENSG00000237346.2; LIRA.1060
B4	26	ENSG00000249790.3; ENSG00000271736.2; ENSG00000285933.1; ENSG00000283633.1; ENSG00000258114.1; ENSG00000224177.8; ENSG00000224259.7; LIRA.181; ENSG00000232265.8; LIRA.1403; ENSG00000251434.1; ENSG00000253819.2; ENSG00000253986.1; ENSG00000277526.5; LIRA.889; LIRA.800; ENSG00000254266.6; ENSG00000225807.2; ENSG00000285486.1; LIRA.262; LIRA.63; LIRA.802; ENSG00000233237.8; ENSG00000250101.1; LIRA.1409; LIRA.182;
B5	17	LIRA.481; LIRA.31; LIRA.33; LIRA.480; ENSG00000230631.1; LIRA.29; LIRA.30; LIRA.38; LIRA.706; LIRA.346; LIRA.1039; LIRA.880; LIRA.616; ENSG00000250634.6; ENSG00000256124.6; LIRA.260; ENSG00000253449.1
B6	87	ENSG00000253474.2, LIRA.1062, LIRA.1057; ENSG00000233236.1; ENSG00000238266.2; LIRA.1058; LIRA.1552; ENSG00000247516.8; LIRA.1111; ENSG00000225172.6; LIRA.1110; ENSG00000250739.1; ENSG00000166770.11; ENSG00000253522.6; ENSG00000228221.6; ENSG00000260455.2; LIRA.888; ENSG00000233410.1; ENSG00000263745.7; LIRA.819; ENSG00000235492.1; ENSG00000249258.2; LIRA.986; LIRA.1291; ENSG00000205837.7; LIRA.223; ENSG00000250241.6; ENSG00000260302.3; LIRA.1319; ENSG00000214049.8; LIRA.983; LIRA.893; LIRA.946; ENSG00000249159.6; ENSG00000234283.1; LIRA.806; LIRA.937; LIRA.1318; LIRA.576; ENSG00000224099.2; ENSG00000249464.6; LIRA.1353; LIRA.1371; ENSG00000238107.1; LIRA.748; LIRA.726; ENSG00000283573.1; LIRA.1080; LIRA.1222; ENSG00000271893.1; LIRA.321; ENSG00000259594.6; ENSG00000272168.8; LIRA.935; ENSG00000234962.6; ENSG00000231533.2; ENSG00000226686.8; ENSG00000233828.5; LIRA.894; LIRA.280; LIRA.282; LIRA.1106; ENSG00000227121.2; ENSG00000259692.6; LIRA.803; ENSG00000229989.4; ENSG00000250173.1; LIRA.216; LIRA.1365; ENSG00000256995.8; LIRA.535; LIRA.310; ENSG00000225579.2; ENSG00000235021.1; ENSG00000228561.2; ENSG00000272070.1; LIRA.222; LIRA.839; ENSG00000267308.3; ENSG00000249364.6; ENSG00000258912.2; ENSG00000236463.1; ENSG00000248125.2; ENSG00000283215.2; LIRA.28; LIRA.123; LIRA.172
B7	67	ENSG00000224739.2; ENSG00000230690.2; ENSG00000249797.2; LIRA.698; ENSG00000224400.5; ENSG00000285354.1; LIRA.1471; ENSG00000259042.3; ENSG00000234177.5; LIRA.700; ENSG00000244620.1; LIRA.484; ENSG00000223930.7; LIRA.695; ENSG00000228876.4; LIRA.1482; ENSG00000232555.2; ENSG00000226777.7; LIRA.211; ENSG00000232444.1; LIRA.175; ENSG00000250334.6; LIRA.1188; LIRA.142; LIRA.1183; ENSG00000260409.1; ENSG00000250891.2; LIRA.166; ENSG00000260876.6; ENSG00000227060.7; ENSG00000255418.6; LIRA.177; ENSG00000215386.13; ENSG00000228484.3; ENSG00000253619.1; LIRA.498; ENSG00000251002.8; LIRA.163; LIRA.1022; LIRA.494; LIRA.1218; ENSG00000274317.2; LIRA.1347; ENSG00000243701.7; ENSG00000248690.8; ENSG00000226581.2; ENSG00000234622.6; ENSG00000237484.6; ENSG00000242258.1; ENSG00000283072.3; LIRA.497; ENSG00000223511.7; ENSG00000233834.6; LIRA.337; LIRA.985; ENSG00000223985.2; ENSG00000251443.2; ENSG00000234215.3; ENSG00000228215.3; ENSG00000261390.6; ENSG00000254416.6; ENSG00000259590.1; ENSG00000270071.3; ENSG00000224184.6; ENSG00000284999.1; ENSG00000226375.1; ENSG00000231439.4
B8	35	ENSG00000260645.2; ENSG00000225948.2; LIRA.764; ENSG00000231081.1; LIRA.743; ENSG00000233154.6; ENSG00000283141.1; LIRA.266; ENSG00000285571.1; ENSG00000230499.1; ENSG00000228139.1; ENSG00000230131.6; ENSG00000223561.7; ENSG00000264695.1; ENSG00000267711.1; LIRA.1197; ENSG00000283317.1; LIRA.315; ENSG00000224950.2; ENSG00000273487.1; ENSG00000232411.1; ENSG00000237166.2; ENSG00000235665.6; ENSG00000256427.2; ENSG00000267130.1; ENSG00000223842.1;

		ENSG00000254343.2; ENSG00000261083.3; ENSG00000235806.1; ENSG00000240996.1; ENSG00000227712.2; ENSG00000285210.1; ENSG00000231509.1; LIRA.1079; ENSG00000256538.2
B9	30	ENSG00000227502.3; ENSG00000259070.7; LIRA.1261; ENSG00000255652.4; ENSG00000198685.4; ENSG00000231829.4; LIRA.1170; ENSG00000230105.1; LIRA.939; ENSG00000254396.1; LIRA.960; LIRA.1150; LIRA.483; ENSG00000260664.2; LIRA.1285; ENSG00000226669.2; LIRA.363; LIRA.365; LIRA.1491; ENSG00000237359.2; ENSG00000283422.1; ENSG00000229727.7; LIRA.944; ENSG00000235731.3; LIRA.1241; LIRA.881; LIRA.1272; LIRA.938; ENSG00000279727.1; ENSG00000249601.2
B10	23	ENSG00000273443.1; LIRA.124; LIRA.592; LIRA.403; LIRA.191; LIRA.407; ENSG00000253227.2; ENSG00000236700.6; LIRA.125; LIRA.825; ENSG00000233081.1; ENSG00000235151.1; LIRA.1086; LIRA.1214; ENSG00000285639.1; ENSG00000262898.3; ENSG00000226954.1; LIRA.196; LIRA.1100; ENSG00000233721.1; LIRA.151; ENSG00000272094.1; LIRA.1362
B11	30	LIRA.1040; ENSG00000248810.2; ENSG00000230387.4; ENSG00000251127.2; LIRA.1030; ENSG00000255801.1; ENSG00000228022.6; ENSG00000249173.6; ENSG00000213373.7; LIRA.636; LIRA.240; LIRA.1461; LIRA.1123; LIRA.1139; ENSG00000248455.6; LIRA.947; LIRA.632; ENSG00000254872.3; ENSG00000253214.2; LIRA.606; LIRA.842; LIRA.987; LIRA.488; ENSG00000251230.6; LIRA.391; ENSG00000223914.4; ENSG00000229891.2; ENSG00000232680.2; LIRA.186; ENSG00000230923.2
B12	13	ENSG00000283646.2; LIRA.909; ENSG00000283445.1; LIRA.278; ENSG00000261804.2; ENSG00000229205.5; ENSG00000180769.10; LIRA.615; ENSG00000258819.2; LIRA.120; ENSG00000253315.1; ENSG00000273172.1; ENSG00000258919.1
B13	40	ENSG00000231106.2; LIRA.396; ENSG00000228065.11; LIRA.236; ENSG00000258018.2; ENSG00000254101.7; ENSG00000239268.3; ENSG00000248703.2; LIRA.366; ENSG00000258710.8; ENSG00000285578.1; ENSG00000249926.2; ENSG00000285079.1; LIRA.254; ENSG00000225930.4; ENSG00000240350.3; ENSG00000267337.2; LIRA.1138; ENSG00000246363.3; LIRA.730; ENSG00000227467.3; ENSG00000266604.1; ENSG00000232310.8; ENSG00000261327.5; LIRA.250; ENSG00000258279.4; ENSG00000280623.1; LIRA.1438; ENSG00000285992.1; ENSG00000259072.2; LIRA.343; LIRA.1432; ENSG00000233208.6; LIRA.1295; ENSG00000258084.6; ENSG00000228157.4; ENSG00000228590.2; ENSG00000222032.1; ENSG00000247317.3; ENSG00000263667.3
B14	45	LIRA.332; ENSG00000249297.2; LIRA.1028; ENSG00000257654.1; ENSG00000257883.1; ENSG00000278090.3; ENSG00000232229.6; ENSG00000204603.7; LIRA.102; LIRA.324; ENSG00000234928.1; ENSG00000237361.3; ENSG00000284237.1; ENSG00000283183.2; ENSG00000251584.2; LIRA.967; LIRA.1243; ENSG00000230731.3; ENSG00000231566.2; ENSG00000189275.4; ENSG00000223466.2; ENSG00000272438.1; ENSG00000239482.6; LIRA.1184; ENSG00000285847.2; ENSG00000230699.2; ENSG00000178248.11; ENSG00000282418.1; LIRA.117; ENSG00000212939.2; LIRA.322; ENSG00000267767.3; ENSG00000223764.2; ENSG00000230400.3; ENSG00000227017.1; LIRA.883; LIRA.89; ENSG00000257239.1; LIRA.81; ENSG00000231482.3; ENSG00000255250.2; ENSG00000262786.1; ENSG00000235126.1; ENSG00000226519.2; ENSG00000250266.2
B15	14	ENSG00000235532.1; ENSG00000280953.2; ENSG00000245954.8; ENSG00000226530.2; ENSG00000256128.6; ENSG00000226423.1; ENSG00000189238.6; ENSG00000230317.2; ENSG00000235576.1; ENSG00000246223.9; ENSG00000249667.1; ENSG00000231682.1; ENSG00000254275.6; LIRA.345
B16	31	ENSG00000251381.8; ENSG00000250986.1; ENSG00000259439.2; LIRA.143; ENSG00000251129.2; ENSG00000214548.18; LIRA.239; ENSG00000267506.5; ENSG00000267466.1; LIRA.1498; LIRA.697; ENSG00000231419.6; LIRA.1363; LIRA.1275; ENSG00000256209.2; ENSG00000241743.4; ENSG00000235994.4; ENSG00000245812.3; ENSG00000275232.1; ENSG00000236502.1; LIRA.1505; LIRA.1299; ENSG00000253301.6; ENSG00000265179.7; ENSG00000225156.2; ENSG00000225746.13; ENSG00000232884.9; ENSG00000225489.7; ENSG00000235142.10; ENSG00000267339.7; ENSG00000269416.6
B17	13	LIRA.679; LIRA.675; LIRA.678; LIRA.670; LIRA.676; LIRA.680; LIRA.668; LIRA.667; LIRA.683; LIRA.691; ENSG00000253364.2; LIRA.669; LIRA.684
B18	136	ENSG00000246100.4, ENSG00000185168.5, ENSG00000231680.1, ENSG00000261222.3, ENSG00000176320.2, ENSG00000228058.2, LIRA.202, ENSG00000231412.2, ENSG00000281162.2, ENSG00000232063.2, ENSG00000261172.1, ENSG00000254952.1, LIRA.741, ENSG00000285662.2, ENSG00000136315.4, LIRA.316, ENSG00000258831.1, ENSG00000274605.2, ENSG00000227508.6, LIRA.971, ENSG00000224397.7, LIRA.319, LIRA.318, LIRA.1454, ENSG00000230138.2, ENSG00000285492.1, ENSG00000265519.1, LIRA.1075, LIRA.796, ENSG00000204960.7, ENSG00000267364.2, ENSG00000246430.7, ENSG00000224307.2, ENSG00000269877.3, ENSG00000262097.2, ENSG00000235478.5, LIRA.1133, ENSG00000285163.1, ENSG00000285040.1, ENSG00000282572.2, LIRA.1242, ENSG00000238042.5, ENSG00000233746.2, LIRA.637, ENSG00000285954.1, ENSG00000229792.1, ENSG00000255666.7, ENSG00000284930.1, LIRA.878, ENSG00000230836.1, ENSG00000249771.2, LIRA.1333, LIRA.1231, ENSG00000232591.2, ENSG00000266088.6, ENSG00000232053.7,

		ENSG00000254639.1, LIRA.392, LIRA.760, LIRA.317, LIRA.563, LIRA.875, LIRA.565, LIRA.746, LIRA.765, ENSG00000227082.3, LIRA.756, ENSG00000237879.1, ENSG00000205056.8, ENSG00000234184.6, LIRA.1134, ENSG00000205300.3, ENSG00000227925.2, LIRA.1052, ENSG00000285630.1, ENSG00000253507.5, ENSG00000255557.1, ENSG00000269667.2, LIRA.1097, ENSG00000214797.3, ENSG00000253177.2, ENSG00000265743.1, ENSG00000259717.1, ENSG00000234506.5, ENSG00000258867.6, LIRA.1342, LIRA.229, ENSG00000258754.8, LIRA.858, LIRA.5, ENSG00000237525.7, ENSG00000250274.2, LIRA.1114, ENSG00000229981.8, LIRA.655, ENSG00000226004.1, ENSG00000203999.9, ENSG00000236914.4, ENSG00000278214.1, ENSG00000255363.3, ENSG00000235888.3, ENSG00000259130.2, ENSG00000236403.2, ENSG00000230649.3, ENSG00000235621.9, ENSG00000231528.3, ENSG00000234572.1, ENSG00000256955.2, LIRA.126, ENSG00000237803.6, LIRA.1418, ENSG00000237797.1, ENSG00000253123.4, ENSG00000257595.3, ENSG00000241316.8, ENSG00000254288.1, ENSG00000187621.15, ENSG00000272908.1, ENSG00000260420.3, ENSG00000229771.2, ENSG00000268804.1, ENSG00000277851.2, ENSG00000225684.4, ENSG00000232539.1, ENSG00000276471.1, ENSG00000225675.2, ENSG00000234390.4, ENSG00000223834.5, ENSG00000259005.1, ENSG00000249236.2, ENSG00000251365.4, LIRA.612, ENSG00000253161.5, ENSG00000229454.1, ENSG00000258689.1, ENSG00000179082.3
B19	101	LIRA.1069, LIRA.1068, LIRA.1072, ENSG00000265369.3, LIRA.1238, ENSG00000247134.7, ENSG00000228742.11, ENSG00000197503.4, LIRA.1502, ENSG00000266976.3, ENSG00000226900.2, ENSG00000271952.2, ENSG00000227066.2, LIRA.1073, LIRA.1067, LIRA.1055, LIRA.195, ENSG00000263677.2, ENSG00000177335.11, LIRA.1146, ENSG00000255980.1, ENSG00000253140.2, ENSG00000253821.2, LIRA.1233, ENSG00000270087.5, LIRA.43, LIRA.272, LIRA.41, LIRA.291, ENSG00000251152.1, LIRA.1324, LIRA.630, ENSG00000285722.1, ENSG00000285108.1, LIRA.1143, ENSG00000230628.1, ENSG00000236834.1, ENSG00000253217.1, ENSG00000226453.2, LIRA.593, ENSG00000231877.1, ENSG00000234464.2, ENSG00000254813.5, ENSG00000232721.2, ENSG00000196758.3, ENSG00000226397.8, ENSG00000205293.5, ENSG00000260430.2, ENSG00000237471.2, ENSG00000273102.1, ENSG00000228495.2, ENSG00000230107.1, LIRA.453, ENSG00000285838.2, LIRA.1145, ENSG00000259420.5, LIRA.639, LIRA.915, ENSG00000224559.2, ENSG00000226856.7, LIRA.94, ENSG00000225127.3, ENSG00000259269.2, ENSG00000246084.5, ENSG00000285624.2, ENSG00000224687.2, ENSG00000234191.1, ENSG00000239941.1, ENSG00000226383.7, ENSG00000276850.5, ENSG00000232977.7, ENSG00000232190.3, ENSG00000231453.1, ENSG00000284430.1, ENSG00000271119.1, ENSG00000226237.2, LIRA.450, LIRA.62, ENSG00000250041.3, LIRA.666, ENSG00000234261.4, LIRA.657, ENSG00000259153.2, LIRA.661, LIRA.439, ENSG00000254802.1, ENSG00000258534.1, ENSG00000256576.2, ENSG00000267123.7, ENSG00000265752.3, ENSG00000258512.1, LIRA.111, ENSG00000214146.3, ENSG00000253298.2, ENSG00000271820.1, ENSG00000248752.3, ENSG00000228044.3, LIRA.660, LIRA.853, ENSG00000229192.6, ENSG00000262185.3
B20	67	ENSG00000225231.2, LIRA.338, LIRA.1192, ENSG00000229855.9, LIRA.1194, ENSG00000235244.4, LIRA.1314, LIRA.435, LIRA.115, ENSG00000239572.3, ENSG00000233922.3, ENSG00000232046.7, ENSG00000253508.1, ENSG00000272808.4, LIRA.113, ENSG00000267325.2, LIRA.112, LIRA.830, LIRA.256, ENSG00000122548.5, LIRA.1091, LIRA.1428, ENSG00000226051.8, LIRA.1417, ENSG00000272692.2, LIRA.255, ENSG00000248360.7, ENSG00000228824.7, ENSG00000231711.2, ENSG00000243144.7, LIRA.375, ENSG00000256001.2, ENSG00000222004.7, ENSG00000214870.9, ENSG00000225087.2, ENSG00000244706.4, ENSG00000226476.6, LIRA.1397, LIRA.283, ENSG00000261645.7, LIRA.341, ENSG00000231010.1, ENSG00000247970.2, ENSG00000255571.9, ENSG00000273297.2, ENSG00000232386.9, ENSG00000175772.11, ENSG00000204241.8, LIRA.1180, ENSG00000256982.2, ENSG00000249574.1, LIRA.1312, ENSG00000234170.6, ENSG00000238078.1, ENSG00000223387.6, ENSG00000229694.7, LIRA.1092, ENSG00000265055.2, ENSG00000233760.2, ENSG00000239467.6, ENSG00000273415.3, ENSG00000204588.5, ENSG00000229557.1, ENSG00000250490.1, ENSG00000267372.2, ENSG00000227683.1, ENSG00000234350.5
B21	149	ENSG00000229807.13, ENSG00000176728.10, ENSG00000231535.7, ENSG00000204792.2, ENSG00000227706.4, ENSG00000251209.9, ENSG00000253853.3, ENSG00000229236.3, LIRA.1088, LIRA.371, ENSG00000206195.11, LIRA.707, LIRA.541, ENSG00000230426.4, ENSG00000223749.11, LIRA.768, ENSG00000227403.2, ENSG00000205611.5, ENSG00000234147.2, ENSG00000254319.6, LIRA.1507, ENSG00000248874.5, ENSG00000247765.2, ENSG00000231131.8, LIRA.378, ENSG00000285534.1, ENSG00000234426.3, LIRA.701, ENSG00000189229.11, LIRA.1557, ENSG00000271584.2, LIRA.538, ENSG00000267374.2, ENSG00000283235.2, ENSG00000130600.19, ENSG00000234665.9, ENSG00000227674.3, ENSG00000236209.1, LIRA.837, ENSG00000276476.3, LIRA.188, ENSG00000258498.8, ENSG00000251555.1, ENSG00000257647.1, LIRA.394, LIRA.1260, LIRA.330, ENSG00000280560.2, LIRA.1413, ENSG00000228709.1, LIRA.1446, LIRA.48, LIRA.1447, ENSG00000264404.3, ENSG00000225493.1, LIRA.930, LIRA.1076, LIRA.1265, LIRA.580, ENSG00000234690.7, ENSG00000231476.1, ENSG00000253967.1, LIRA.1558, LIRA.224, ENSG00000237531.6, LIRA.1152, ENSG00000234944.1, LIRA.86, ENSG00000235578.1, LIRA.745, ENSG00000230490.3, ENSG00000215808.4,

	LIRA.1378, ENSG00000229660.1, ENSG00000223342.2, ENSG00000284240.1, ENSG00000229484.1, LIRA.136, LIRA.812, ENSG00000283458.1, LIRA.647, ENSG00000267780.2, ENSG00000269994.3, ENSG00000267568.6, ENSG00000267924.1, LIRA.744, ENSG00000263958.2, ENSG00000245694.10, LIRA.1220, ENSG00000223855.2, LIRA.648, ENSG00000258517.1, LIRA.386, ENSG00000256783.1, ENSG00000267501.1, ENSG00000227107.1, ENSG00000277128.2, ENSG00000262585.1, LIRA.788, ENSG00000248187.1, ENSG00000267521.1, LIRA.1293, ENSG00000223486.1, ENSG00000237422.2, LIRA.1189, ENSG00000227888.4, LIRA.847, ENSG00000257817.2, LIRA.10, ENSG00000226159.2, ENSG00000229628.1, ENSG00000237357.2, ENSG00000237372.4, ENSG00000285867.1, ENSG00000253230.9, ENSG00000259218.6, ENSG00000231826.6, LIRA.738, LIRA.87, ENSG00000260289.2, ENSG00000258807.5, ENSG00000270816.6, LIRA.659, ENSG00000230563.4, ENSG00000248991.1, LIRA.1102, LIRA.618, ENSG00000253395.1, LIRA.1025, LIRA.1029, ENSG00000280018.4, ENSG00000185433.10, ENSG00000250658.1, ENSG00000237980.2, ENSG00000224137.1, ENSG00000250501.3, ENSG00000282965.1, ENSG00000256424.2, ENSG00000237609.1, ENSG00000259664.3, ENSG00000270279.1, ENSG00000250415.1, LIRA.251, ENSG00000267414.1, ENSG00000228692.2, ENSG00000235728.1, ENSG00000284624.1, ENSG00000250974.4, ENSG00000249241.1

Table S9. Correlation (upper) and p-value (lower) for each WGCNA module in the pediatric versus adult comparison.

	CBFB::MYH11		CEBPA		KMT2A-R		FLT3-ITD		RUNX1::RUNX1T1	
	Adult	Pediatric	Adult	Pediatric	Adult	Pediatric	Adult	Pediatric	Adult	Pediatric
B1	-0.1858	-0.0775	-0.1617	0.1316	-0.1480	0.3450	-0.0664	0.0465	-0.0637	0.0827
B2	-0.0873	-0.0271	-0.1811	-0.1294	-0.2410	0.0897	0.3368	0.3165	-0.1118	-0.0527
B3	-0.3428	-0.3361	-0.0174	-0.0583	0.0834	0.3274	0.1592	0.3883	-0.1659	-0.1812
B4	-0.2553	-0.2045	0.3014	0.3092	-0.1764	0.0948	-0.0556	0.0116	-0.1025	-0.0272
B5	-0.0938	-0.1618	0.2718	0.3033	-0.0460	0.1315	-0.0948	0.0722	-0.3022	-0.2612
B6	-0.0741	-0.1189	0.4734	0.3402	-0.3459	-0.2432	-0.1443	-0.1478	0.1960	0.1059
B7	-0.1031	-0.0398	0.3607	0.1924	-0.3936	-0.2268	0.1833	0.2337	-0.1632	-0.1414
B8	-0.0023	-0.2762	0.2114	0.0092	0.0074	-0.0386	0.0754	-0.0160	0.1533	-0.1098
B9	-0.1437	-0.3727	0.2723	0.1091	-0.1140	0.0199	0.0442	0.0565	0.0476	0.0454
B10	0.0462	-0.0956	-0.1639	0.0084	0.0460	0.1847	0.0072	0.0461	-0.1792	0.0145
B11	0.2556	0.1063	-0.1858	-0.0532	-0.1395	0.1378	-0.0652	-0.0317	-0.0849	0.0051
B12	-0.0007	-0.0511	0.0331	0.1602	-0.1822	0.0538	-0.0834	-0.1172	0.1071	0.0778
B13	0.5724	0.3465	-0.2112	-0.1651	-0.2661	-0.1116	-0.0636	-0.1276	0.0262	0.0303
B14	0.3562	0.2333	0.0231	-0.0378	-0.3959	-0.3605	-0.0599	-0.1910	0.3163	0.2743
B15	0.0629	-0.1128	-0.0310	-0.0176	-0.0440	0.1824	-0.0873	-0.1227	0.0548	0.1015
B16	-0.1762	-0.2136	-0.0157	-0.0256	-0.0917	0.0782	-0.1490	-0.1809	0.5376	0.4127
B17	0.0230	-0.0173	0.0594	0.0246	-0.0573	0.0453	-0.0947	-0.0779	0.0853	0.0212
B18	0.2560	0.0372	-0.2229	-0.1595	0.2382	0.3547	-0.1034	-0.1612	-0.1162	-0.1989
B19	-0.0788	-0.2121	-0.1897	-0.1540	0.5157	0.5334	-0.1446	-0.1692	-0.0404	-0.1132
B20	-0.2444	-0.2455	-0.2314	-0.1707	0.3961	0.5669	0.0310	0.0860	-0.1566	-0.1491
B21	0.0926	-0.2011	-0.1303	-0.0491	0.0402	0.3250	-0.0698	-0.1270	0.0678	0.0111

	CBFB::MYH11		CEBPA		KMT2A-R		FLT3-ITD		RUNX1::RUNX1T1	
	Adult	Pediatric	Adult	Pediatric	Adult	Pediatric	Adult	Pediatric	Adult	Pediatric
B1	8.2E-04	1.7E-01	3.7E-03	1.8E-02	7.9E-03	2.1E-10	2.4E-01	4.1E-01	2.6E-01	1.4E-01
B2	1.2E-01	6.3E-01	1.1E-03	2.0E-02	1.3E-05	1.1E-01	5.9E-10	6.7E-09	4.5E-02	3.5E-01
B3	2.8E-10	6.5E-10	7.6E-01	3.0E-01	1.4E-01	1.9E-09	4.2E-03	5.4E-13	2.9E-03	1.1E-03
B4	3.6E-06	2.3E-04	3.6E-08	1.5E-08	1.5E-03	9.0E-02	3.2E-01	8.4E-01	6.7E-02	6.3E-01
B5	9.3E-02	3.7E-03	7.7E-07	2.9E-08	4.1E-01	1.8E-02	9.0E-02	2.0E-01	3.3E-08	2.1E-06
B6	1.9E-01	3.3E-02	2.5E-19	3.9E-10	1.9E-10	1.0E-05	9.6E-03	8.0E-03	4.1E-04	5.8E-02
B7	6.5E-02	4.8E-01	2.7E-11	5.3E-04	2.4E-13	4.1E-05	9.7E-04	2.3E-05	3.4E-03	1.1E-02
B8	9.7E-01	5.0E-07	1.4E-04	8.7E-01	8.9E-01	4.9E-01	1.8E-01	7.8E-01	5.9E-03	4.9E-02
B9	9.9E-03	5.1E-12	7.3E-07	5.1E-02	4.1E-02	7.2E-01	4.3E-01	3.1E-01	4.0E-01	4.2E-01
B10	4.1E-01	8.7E-02	3.2E-03	8.8E-01	4.1E-01	8.8E-04	9.0E-01	4.1E-01	1.3E-03	8.0E-01
B11	3.5E-06	5.7E-02	8.2E-04	3.4E-01	1.2E-02	1.3E-02	2.4E-01	5.7E-01	1.3E-01	9.3E-01
B12	9.9E-01	3.6E-01	5.5E-01	4.0E-03	1.0E-03	3.4E-01	1.4E-01	3.6E-02	5.5E-02	1.6E-01
B13	2.4E-29	1.8E-10	1.4E-04	3.0E-03	1.3E-06	4.6E-02	2.6E-01	2.2E-02	6.4E-01	5.9E-01
B14	4.9E-11	2.4E-05	6.8E-01	5.0E-01	1.7E-13	2.8E-11	2.8E-01	5.8E-04	6.9E-09	6.0E-07
B15	2.6E-01	4.4E-02	5.8E-01	7.5E-01	4.3E-01	1.0E-03	1.2E-01	2.8E-02	3.3E-01	6.9E-02
B16	1.5E-03	1.2E-04	7.8E-01	6.5E-01	1.0E-01	1.6E-01	7.5E-03	1.1E-03	1.9E-25	1.2E-14
B17	6.8E-01	7.6E-01	2.9E-01	6.6E-01	3.1E-01	4.2E-01	9.0E-02	1.6E-01	1.3E-01	7.1E-01
B18	3.4E-06	5.1E-01	5.6E-05	4.2E-03	1.6E-05	6.0E-11	6.4E-02	3.8E-03	3.7E-02	3.4E-04
B19	1.6E-01	1.3E-04	6.3E-04	5.7E-03	3.3E-23	5.3E-25	9.5E-03	2.4E-03	4.7E-01	4.3E-02
B20	9.5E-06	8.6E-06	2.8E-05	2.1E-03	1.7E-13	1.1E-28	5.8E-01	1.2E-01	4.9E-03	7.4E-03
B21	9.8E-02	2.9E-04	2.0E-02	3.8E-01	4.7E-01	2.5E-09	2.1E-01	2.3E-02	2.3E-01	8.4E-01

P-values above 0.01 are shown grey.

Supplementary references

1. Severens JF, Karakaslar EO, van der Reijden BA, Sanchez-Lopez E, van den Berg RR, Halkes CJM, et al. Mapping AML heterogeneity - multi-cohort transcriptomic analysis identifies novel clusters and divergent ex-vivo drug responses. Leukemia. 2024;38(4):751–761.
2. Zheng J, Song Y, Li Z, Tang A, Fei Y, He W. The implication of lncRNA expression pattern and potential function of lncRNA RP4-576H24.2 in acute myeloid leukemia. Cancer Med. 2019;8(17):7143–7160.
3. Morlando M, Ballarino M, Fatica A. Long Non-Coding RNAs: New Players in Hematopoiesis and Leukemia. Frontiers in medicine. 2015;2:23.
4. Altieri F, Buono L, Lanzilli M, Mirabelli P, Cianflone A, Beneduce G, et al. LINC00958 as new diagnostic and prognostic biomarker of childhood acute lymphoblastic leukaemia of B cells. Front Oncol. 2024;14:1388154.
5. Connerty P, Moles E, de Bock CE, Jayatilleke N, Smith JL, Meshinchi S, et al. Development of siRNA-Loaded Lipid Nanoparticles Targeting Long Non-Coding RNA LINC01257 as a Novel and Safe Therapeutic Approach for t(8;21) Pediatric Acute Myeloid Leukemia. Pharmaceutics. 2021;13(10):1681.
6. Li R, Wu S, Wu X, Zhao P, Li J, Xue K, et al. Immune-relatedlncRNAs can predict the prognosis of acute myeloid leukemia. Cancer Medicine. 2022;11(3):888–899.
7. Zhang M, Zhang LL, Yi LB, Tu XN, Zhou Y, Li DY, et al. Comprehensive analysis of immune-related lncRNAs in AML patients uncovers potential therapeutic targets and prognostic biomarkers. Heliyon. 2024;10(9):e30616.
8. Shi K, Li D, Peng BH, Guo Q. The high-risk model associated with SYTL4 predicts poor prognosis and correlates with immune infiltration in AML. Biochem Biophys Rep. 2024;41:101859.
9. Gasic V, Karan-Djurasevic T, Pavlovic D, Zukic B, Pavlovic S, Tosic N. Diagnostic and Therapeutic Implications of Long Non-Coding RNAs in Leukemia. Life. 2022;12(11):1770.
10. Zhang J, Griffith M, Miller CA, Griffith OL, Spencer DH, Walker JR, et al. Comprehensive discovery of noncoding RNAs in acute myeloid leukemia cell transcriptomes. Exp Hematol. 2017;55:19–33.
11. Verma D, Kapoor S, Kumari S, Sharma D, Singh J, Benjamin M, et al. Decoding the genetic symphony: Profiling protein-coding and long noncoding RNA expression in T-acute lymphoblastic leukemia for clinical insights. PNAS Nexus. 2024;3(2):pgae011.
12. Melo CP, Campos CB, Rodrigues Jde O, Aguirre-Neto JC, Atalla A, Pianovski MA, et al. Long non-coding RNAs: biomarkers for acute leukaemia subtypes. Br J Haematol. 2016;173(2):318–320.
13. Lobo-Alves SC, Oliveira LA, Kretzschmar GC, Valengo AE, Rosati R. Long noncoding RNA expression in acute lymphoblastic leukemia: A systematic review. Crit Rev Oncol Hematol. 2024;196:104290.
14. Zhu Y, Jian J, Niu Y, Yang X, Guo Y, Zhao L, et al. Construction and validation of an NAD + metabolism-related lncRNA signature for predicting the prognosis and immune landscape of acute myeloid leukemia. Hematology. 2023;28(1):2231760.
15. Zhang J, Zhang H, Wang X, Zhao Y, Fu Y, Liu X. PCAT18, as a novel differentially regulated long noncoding RNA in adult acute myeloid leukemia patients revealed by next-generation sequencing. Int J Lab Hematol. 2020;42(6):858–865.
16. Yu P, Lan H, Song X, Pan Z. High Expression of the SH3TC2-DT/SH3TC2 Gene Pair Associated With FLT3 Mutation and Poor Survival in Acute Myeloid Leukemia: An Integrated TCGA Analysis. Front Oncol. 2020;10:829.
17. Dong X, Xu X, Guan Y. LncRNA LINC00899 promotes progression of acute myeloid leukaemia by modulating miR-744-3p/YY1 signalling. Cell Biochem Funct. 2020;38(7):955–964.
18. Wang Y, Li Y, Song HQ, Sun GW. Long non-coding RNA LINC00899 as a novel serum biomarker for diagnosis and prognosis prediction of acute myeloid leukemia. Eur Rev Med Pharmacol Sci. 2018;22(21):7364–7370.
19. Arab I, Lim SG, Suk K, Lee WH. LINC01270 Regulates the NF-kappaB-Mediated Pro-Inflammatory Response via the miR-326/LDOC1 Axis in THP-1 Cells. Cells. 2024;13(23):2027.
20. Zia S, Rehman N, Ejaz S, Shahid M, Ali M, Shahid R. Transcriptomic and in silico analysis of BLACE (B-cell acute lymphoblastic leukemia expressed), a new non-coding RNA, as a diagnostic biomarker in B-cell ALL. Int J Biochem Cell Biol. 2024;177:106698.
21. Lai X, Wei J, Gu XZ, Yao XM, Zhang DS, Li F, et al. Dysregulation of LINC00470 and METTL3 promotes chemoresistance and suppresses autophagy of chronic myelocytic leukaemia cells. J Cell Mol Med. 2021;25(9):4248–4259.

22. Yan JH, Yao L, Tan Y, Wang Y. LINC00504 promotes the progression of acute myeloid leukemia by targeting MDM2. Neoplasma. 2023;70(2):199–207.
23. Harris RA, Stevens JM, Pickering DL, Althof PA, Smith LM, Sanmann JN, et al. Frequency, variations, and prognostic implications of chromosome 14q32 deletions in chronic lymphocytic leukemia. Leuk Res. 2021;110:106665.
24. Huang M, Zheng J, Ren Y, Zhu J, Kou L, Nie J. LINC00221 suppresses the malignancy of children acute lymphoblastic leukemia. Biosci Rep. 2020;40(5):BSR20194070.
25. Connerty P, Lock RB. The tip of the iceberg—The roles of long noncoding RNAs in acute myeloid leukemia. WIREs RNA. 2023;14(6):e1796.
26. Yin Z, Shen H, Gu CM, Zhang MQ, Liu Z, Huang J, et al. MiRNA-142-3P and FUS can be Sponged by Long Noncoding RNA DUBR to Promote Cell Proliferation in Acute Myeloid Leukemia. Front Mol Biosci. 2021;8:754936.
27. Sellers ZP, Bolkun L, Kloczko J, Wojtaszewska ML, Lewandowski K, Moniuszko M, et al. Increased methylation upstream of the MEG3 promotor is observed in acute myeloid leukemia patients with better overall survival. Clin Epigenetics. 2019;11(1):50.
28. Benetatos L, Hatzimichael E, Dasoula A, Dranitsaris G, Tsiara S, Syrrou M, et al. CpG methylation analysis of the MEG3 and SNRPN imprinted genes in acute myeloid leukemia and myelodysplastic syndromes. Leuk Res. 2010;34(2):148–153.
29. Lyu Y, Lou J, Yang Y, Feng J, Hao Y, Huang S, et al. Dysfunction of the WT1-MEG3 signaling promotes AML leukemogenesis via p53-dependent and -independent pathways. Leukemia. 2017;31(12):2543–2551.
30. Li J, Li Z, Bai X, Chen X, Wang M, Wu Y, et al. LncRNA UCA1 Promotes the Progression of AML by Upregulating the Expression of CXCR4 and CYP1B1 by Affecting the Stability of METTL14. J Oncol. 2022;2022:2756986. **Correction in:** *J. Oncol.* **2025**, *2025*, 9842168.
31. Guo C, Ju QQ, Zhang CX, Gong M, Li ZL, Gao YY. Overexpression of HOXA10 is associated with unfavorable prognosis of acute myeloid leukemia. BMC Cancer. 2020;20(1):586.
32. Zhai H, Zhao J, Pu J, Zhao P, Wei J. LncRNA-DUXAP8 Regulation of the Wnt/beta-Catenin Signaling Pathway to Inhibit Glycolysis and Induced Apoptosis in Acute Myeloid Leukemia. Turk J Haematol. 2021;38(4):264–272.
